# Genomic Characterization of *Arcobacter butzleri* Isolated From Shellfish: Novel Insight Into Antibiotic Resistance and Virulence Determinants

**DOI:** 10.3389/fmicb.2019.00670

**Published:** 2019-04-16

**Authors:** Francesca Fanelli, Angela Di Pinto, Anna Mottola, Giuseppina Mule, Daniele Chieffi, Federico Baruzzi, Giuseppina Tantillo, Vincenzina Fusco

**Affiliations:** ^1^Institute of Sciences of Food Production (CNR-ISPA), National Research Council of Italy, Bari, Italy; ^2^Department of Veterinary Medicine, University of Bari Aldo Moro, Bari, Italy; ^3^Institute of Biomembranes, Bioenergetics and Molecular Biotechnologies (CNR-IBIOM), National Research Council of Italy, Bari, Italy

**Keywords:** *Aliarcobacter butzleri*, *Arcobacter butzleri*, antibiotic and heavy metal resistance, virulence genes, genomics, food safety, emerging foodborne pathogen, shellfish

## Abstract

*Arcobacter (A.) butzleri* is an emerging pathogenic microorganism, whose taxonomy has been recently suggested to be emended to the *Aliarcobacter (Al.) butzleri* comb. nov. Despite extensive taxonomic analysis, only few fragmented studies have investigated the occurrence and the prevalence of virulence and antibiotic resistance determinants of this species in strains isolated from shellfish. Herein we report for the first time the whole genome sequencing and genomic characterization of two *A. butzleri* strains isolated from shellfish, with particular reference to the antibiotic, heavy metals and virulence determinants. This study supported the taxonomic assignment of these strains to the *Al. butzleri* species, and allowed us to identify antibiotic and metal resistance along with virulence determinants, also additional to those previously reported for the only two *A. butzleri* strains from different environments genomically characterized. Moreover, both strains showed resistance to β-lactams, vanocomycin, tetracycline and erythromycin and susceptibility to aminoglycosides and ciprofloxacin. Beside enlarging the availability of genomic data to perform comparative studies aimed at correlating phenotypic differences associated with ecological niche and geographic distribution with the genetic diversity of *A. butzleri* spp., this study reports the endowment of antibiotic and heavy metal resistance and virulence determinants of these shellfish-isolated strains. This leads to hypothesize a relatively high virulence of *A. butzleri* isolated from shellfish and prompt the need for a wider genomic analysis and for *in vitro* and *in vivo* studies of more strains isolated from this and other ecological niches, to unravel the mechanism of pathogenicity of this species, and the potential risk associated to their consumption.

## Introduction

The current validated taxonomy places the *Arcobacter* genus within the *Campylobacteraceae* family (belonging to the class *Epsilonproteobacteria* of the phylum *Proteobacteria*) together with *Campylobacter* and *Sulfurospirillum* genera. Recently, based on a wide comparative genomic analysis, Waite et al. (2017) proposed the re-classification of the class *Epsilonproteobacteria* with the *Arcobacter* genus ascribed to the *Arcobacteraceae* fam. nov. (type genus: *Arcobacter*, order: *Campylobacterales*, class: *Campylobacteria*, class. nov., phylum: *Campylobacteraeota* phyl. nov.) (Waite et al., 2017, 2018).

*Arcobacter* spp. have a wide diversity of hosts and habitats, with water being one of the main routes of transmission (Collado and Figueras, 2011). *Arcobacter* spp. have been indeed detected in environmental water sources, including rivers, lakes, sewage and plankton (Kim et al., 2010; Levican et al., 2013; Park et al., 2016), marine, domestic (Merga et al., 2014), and drinking water (Jalava et al., 2014; Talay et al., 2016), water distribution pipes (Phillips, 2001), groundwater (Fong et al., 2007), and recreational water (Lee et al., 2012). Four species (namely, *A. butzleri*, *A.* *cryaerophilus*, *A.* *thereius*, and *A.* *skirrowii*) have also been isolated in humans and animals (Banting and Figueras Salvat, 2017) and are able to cause human bacteraemia, endocarditis, peritonitis, gastroenteritis, and diarrhea (Jiang et al., 2010; Figueras et al., 2014; Arguello et al., 2015; Ferreira et al., 2016).

To date this genus includes 26 species (Ramees et al., 2017; Pérez-Cataluña et al., 2018a), which inhabit various ecological niches (Collado and Figueras, 2011; Ferreira et al., 2016). Recently, Pérez-Cataluña et al. (2018a) used a polyphasic approach to revisit the taxonomy of the genus. By setting specific cut-off values for each method (identity of 16S rRNA, genomic indexes such as ANI, AAI, and DDH, multilocus sequence analysis, etc.), they delimited genomic and phylogenetic groups and defined six different genera including *Aliarcobacter* gen. nov. This genus comprises seven species including also *Aliarcobacter (Al.) butzleri* comb. nov., whose type strain was confirmed to be LMG 10828^T^ (ATCC 49616^T^; RM4018; Miller et al., 2007), and the species description as the one given by Vandamme et al. (1992) and Pérez-Cataluña et al. (2018a,b).

However, it must be considered that both the species description of *A. butzleri* and *Al. butzleri* comb. nov., were obtained by analyzing an exiguous number of *A. butzleri* strains (12 by Vandamme et al., 1992 and only two by Pérez-Cataluña et al., 2018a, respectively). Thus, it might be expected that the likely increasing availability of *A. butzleri* genomic sequences could lead this species to a further redefinition.

Recent outcomes and progress related to the pathogenicity of *A. butzleri* have led to the allocation of this species in the list of microbes considered a serious hazard to human health by the International Commission on Microbiological Specifications for Foods (ICMSF, 2002). *A. butzleri* is considered an emerging food-borne enteropathogen and, in recent years, has been associated with enteritis, severe diarrhea, bacteraemia, and septicaemia in humans and enteritis, mastitis, abortion, and stillbirth in cows, sheep, and pigs (Engberg et al., 2000; Lau et al., 2002; Collado and Figueras, 2011; Collado et al., 2014; Ferreira et al., 2014a,b, 2016; Figueras et al., 2014; Van den Abeele et al., 2014; Heimesaat et al., 2015; Gölz et al., 2016; Webb et al., 2016; Franz et al., 2018; Fusco et al., 2018; Flynn et al., 2019). Enteritis due to the ingestion of food contaminated with *Arcobacter* spp. can be self-limiting. Nevertheless, the severity and protraction of the symptoms might require an antibiotic treatment, which might be affected by the (multiple) antibiotic resistance of the strain, thus complicating the treat of the relevant infections. *A. butzleri* is the most prevalent species of this genus in meat products (chicken, pork, beef, lamb), milk, cheese, and shellfish (Figueras et al., 2011a,b; Patyal et al., 2011; Shah et al., 2012; Hausdorf et al., 2013; Lee and Choi, 2013; Rahimi, 2014; Ramees et al., 2014; Lehmann et al., 2015; Ferreira et al., 2016). The presence and persistence in these niches, also endowed by the ability to form biofilms (Ferreira et al., 2013; Girbau et al., 2017), favor its spread and transmission to shellfish and farm animal, and increase the risk associated with food consumption. Although few fragmented studies have been carried out to assess the occurrence of this species in shellfish, *A. butzleri* has been found as the most common species in bivalve molluscs (mussels, clams, oysters, etc.) (Nieva-Echevarria et al., 2013; Levican et al., 2014; Mottola et al., 2016; Leoni et al., 2017). This is most likely due to capture by the filter feeding process of bivalves and to a fecal contamination of the relevant environment, so that *Escherichia coli* has been proposed by Leoni et al. (2017) as an index organism for *A. butzleri* contamination in bivalve molluscs, leading to suggest that these shellfishes could be a reservoir of *A. butzleri* (Leoni et al., 2017). For this reason, consumption of contaminated shellfish, especially if raw or undercooked, which is still a widespread practise (Schauer Weissfeld, 2014), may be source of *A. butzleri* infections in humans. The ability to survive in different environments, the endowment of antibiotic resistance genes and virulence potential found by genomic approaches (Miller et al., 2007; Pérez-Cataluña et al., 2018a), as well as the genetic plasticity conferred by the presence of mobile elements, which allow the transfer of genes (Douidah et al., 2014), are important determinants for the evolution and the fitness of this and the other species of this genus.

As far as we know, extensive genome-based characterization of the species has been carried out only on two *A. butzleri* strains, namely, RM4018 (Culture collection n. LMG 10828^T^), isolated from human feces, and ED-1 isolated from microbial fuel cells (Miller et al., 2007; Pérez-Cataluña et al., 2018a). Considering that contaminated shellfish may be source of *A. butzleri* infection and given that the prevalence and expression of putative virulence and antibiotic resistance genes within this species may vary with the source of the strain (Douidah et al., 2012; Ferreira et al., 2014b; Girbau et al., 2015; Zacharow et al., 2015a), herein we report the antibiotic susceptibility and genomic-based characterization of two *A. butzleri* strains isolated from shellfish, with particular reference to the genetic determinants of the above mentioned traits of pathogenicity.

## Materials and Methods

### Strains and Culture Condition

*Arcobacter butzleri* (Ab) strains 6V and 55 were isolated on 2016 from clams (*Tapes philippinarum*) (Mottola et al., 2016) and mussels (*Mytilus galloprovincialis*) obtained from local fish market in the Apulia region (Italy). These strains were previously identified and typed by MLST (Mottola et al., 2016; Mottola, 2017). Allelic profiles and sequences are available on the *Arcobacter* MLST database ^1^ under the ID numbers 717 (*Ab 6V*) and 839 (*Ab 55*).

Pure cultures were isolated and maintained in the microbial collection of the Institute of Sciences of Food Production, CNR, Bari ^2^. Bacterial strains were maintained at -80°C as pure stock cultures in Brain Heart Infusion (BHI) broth (Oxoid S.p.A., Rodano, Milan, Italy) supplemented with glycerol (30% vol/vol). Cultures were streaked on Agar blood plates (Oxoid, Milan, Italy) and grown at 37°C for 48 h. Working cultures were obtained growing a single colony in 20 mL of BHI broth with 0.6% yeast extract (BHI-YE), at 37°C for 48 h.

### Genome Sequencing and Assembly

DNA isolation was performed by using the Wizard^®^ Genomic DNA Purification Kit (Promega), as previously described by Ercolini et al. (2005). The integrity, purity, and quantity of DNA were assessed as previously described by Fusco et al. (2011), by agarose gel electrophoresis, by NanoDrop-2000 (Thermo Fisher Scientific, Wilmington, DE, United States), and by Qubit 3.0 fluorometer (Life Technologies). DNA was then subjected to whole genome shotgun sequencing using the Ion S5^TM^ library preparation workflow (Thermo Fisher Scientific, Waltman, MA, United States); 400 bp mate-paired reads were generated on the Ion S5^TM^ System (Thermo Fisher Scientific). Duplicate reads were removed by FilterDuplicates (v5.0.0.0) Ionplugin. *De novo* assembly was performed by AssemblerSpades (v.5.0) Ionplugin^TM^.

### Bioinformatic Methods

Genes were predicted and annotated using PROKKA pipeline implemented in the Galaxy platform (Galaxy Tool Version 1.0.0; Afgan et al., 2016). The predicted proteins were submitted to the PFAM annotator tool within the Galaxy platform in order to predict the pfam domains. Protein ID used in the manuscript indicated those obtained by NCBI (National Center for Biotechnology Information) Prokaryotic Genome Annotation Pipeline (Tatusova et al., 2016).

Predicted proteins were assigned to Clusters of Orthologous Groups (COG) functional categories by Web CD-Search Tool (Marchler-Bauer et al., 2017) using an Expected value threshold of 0.01. COG ID were then manually mapped into functional categories^3^.

All the proteins sequences used in this study were retrieved from GenBank (NCBI). The homology-based relationship of *Ab* 55 and *Ab* 6V predicted proteins toward selected proteins was determined by BLASTP algorithm on the NCBI site^4^. Gene models were manually determined, and clustering and orientation were subsequently deduced for the closely linked genes.

Antibiotic resistance genes were predicted by BLASTP search against the Antibiotic Resistance genes Database (ARDB; Liu and Pop, 2009) and beta lactamase database (Naas et al., 2017). Genes associated with antibiotic resistance were also retrieved by keywords terms search within UniProtID entry list obtained by functional annotation.

Functional annotation, subsystem prediction, and metabolic reconstruction comparison were also performed using the RAST server (Aziz et al., 2008). Genes involved in the mechanism of resistance to heavy metals were retrieved by Poole (2017) and used as queries for BLASTP search against *Ab* 55 and *Ab* 6V proteomes.

Genetic divergence was calculated by the ANI/AAI calculator (Goris et al., 2007; Rodriguez-R and Konstantinidis, 2016) which estimates the average nucleotide/aminoacid identity (ANI/AAI) using both best hits (one-way ANI) and reciprocal best hits (two-way ANI) between genomic datasets. The Genome-to-Genome Distance Calculator (GGDC) (Meier-Kolthoff et al., 2013, 2014) web service was used to report DDH for the accurate delineation of prokaryotic subspecies and to calculate differences in G+C genomic content (available at ggdc.dsmz.de). Formula 2 alone was used for analysis, providing an estimation of DDH independent from genome lengths, as recommended by the authors of GGDC for use with any incomplete genomes (Auch et al., 2010; Meier-Kolthoff et al., 2013).

### Antimicrobial Susceptibility Testing

The antimicrobial susceptibility tests for *A. butzleri* isolates were performed by disk diffusion and broth microdilution methods. The disk diffusion test was performed as described by Rathlavath et al. (2017) with modifications. Briefly, *A. butzleri* isolates were grown in 20 ml of BHI broth (Oxoid, United Kingdom) amended with 0.6% yeast extract (YE) (Biolife srl, Italy) under static condition for 48 h at 37°C and then subcultured at 1% in BHI-YE broth and incubated at 37°C for 48 h. Microbial cells were recovered after centrifugation (16,000 rcf × 6 min), washed in sterile 0.9% NaCl solution, adjusting optical density (600 nm) to 0.5. One hundred microliters of this cell suspension were then plated on 4 mm thick cation adjusted Muller Hinton agar (MHIIA, Liofilchem, Italy).

Antibiotic disks, soaked with ampicillin (10 μg/disk), cefotaxime (30 μg/disk), chloramphenicol (30 μg/disk), ciprofloxacin (5 μg/disk), erythromycin (15 μg/disk), gentamicin (10 μg/disk), kanamycin (30 μg/disk), nalidixic acid (30 μg/disk), streptomycin (10 μg/disk), tetracycline (30 μg/disk), vancomycin (30 μg/disk), and penicillin G (10 units/disk) (Biolab Zrt., Hungary), were placed onto inoculated plates and incubated at 37°C under microaerophilic atmosphere (CampyGenTM Compact, Oxoid, United Kingdom), as recommended by BCCM/LMG Bacteria Collection^5^ for *A. butzleri* type strain LMG10828 (RM4018). After 48 h of incubation, inhibition zone diameters were recorded.

For those antibiotics which the tested *A. butzleri* strains did not provide inhibition zone at all, the minimal inhibitory concentration (MIC) was calculated by broth microdilution method as described by Riesenberg et al. (2017). After 48 h incubation at 37°C, microtitre plates were read spectrophotometrically at 600 nm using Varioskan Flash (Thermo Fisher Scientific, United States). To determine minimal bactericidal concentration (MBC), 10 μl of broth from three replicate of all wells without microbial growth was combined in a single sample spotted on MHIIA (Liofilchem, Italy) and incubated as described above up to 72 h. Since no breakpoint values are available for *Arcobacter* spp., classification of strains as susceptible (S), resistant (R), or intermediate (I) was defined according to zone diameter and MIC interpretive standards for *Staphylococcus* spp. (erythromycin, penicillin, and vancomycin) and *Enterobacteriaceae* (ampicillin, gentamicin, cefotaxime, ciprofloxacin, tetracycline, chloramphenicol, nalidixic acid, kanamycin, and streptomycin) (CLSI, 2015), as also reported by Elmali and Can (2017).

The *A.* *butzleri* LMG 10828^T^ (RM4018) strain was used for comparison purposes.

## Results and Discussion

### General Features of *A. butzleri* 6V and 55 Genomes

*Ab* 55 and *Ab* 6V genomes were sequenced using a whole genome shotgun approach on an Ion S5^TM^ platform (Thermo Fisher Scientific) generating around 671,363 and 596,333 reads with a median length of 317 and 320 bp, respectively (Table 1). Genomes were assembled using the Spades v5.0 software for a total of 32 and 46 large contigs (>500 bp) and a GC% of 26.79 and 26.85, respectively. The overall contiguity of the assembly is good, with a N50 of 211 and 129 kbp for *Ab* 55 and *Ab* 6V, respectively; the longest assembled fragment is 403 kbp in length for *Ab* 55 and 251 kbp for *Ab* 6V (performed by QUAST, available at http://quast.sourceforge.net/quast) while the total length of the assembly was around of 2.3 Mb for both genomes. These Whole Genome Shotgun projects have been deposited at DDBJ/ENA/GenBank under the accessions QXMK00000000 (*Ab* 55) and QXNB0000000 (*Ab* 6V). The versions described in this paper are QXMK01000000 (*Ab* 55) and QXNB01000000 (*Ab* 6V).

**Table 1:** Summary of *A. butzleri* 55 and 6V genome sequencing and assembly results.

	*A. butzleri* 55	*A. butzleri* 6V
Total sequenced bases	212,850,706	190,979,223
Mean read length	317	320
Total length	2,330,339	2,303,554
Number of scaffolds	47	61
Largest contig	403,569	251,748
Number reads	671,363	596,833
N50 reads	211,847	129,283
Genome size	2,325,213	2,297,763
GC content	26.79	26.85
Predicted genes	2395	2338
CDS	2344	2289
tRNA	46	44
ncRNA	2	2
rRNA	1, 1, 1 (5S, 16S, 23S)	1, 1, 1 (5S, 16S, 23S)


### Genomic Analysis

The *in silico* MLST of the housekeeping genes retrieved from genomic sequences, confirmed *in vitro* results achieved by Mottola (2017), revealing that *Ab* 55 and *Ab* 6V belong to two novel sequence types, namely, ST675 and ST537, respectively, as they harbor 6/7 and 3/7 new alleles, respectively (Table 2).

**Table 2:** Allelic profiles of *A. butzleri* isolates.

				MLST							
ID	Isolate	Species	Source	*asp*A	*atp*A	*gln*A	*glt*A	*gly*A	*pgm*	*tkt*	ST
717	6V	*Arcobacter butzleri*	clam	**236**	**161**	1	**183**	**521**	**296**	**207**	**537**
839	55	*Arcobacter butzleri*	mussel	**332**	8	1	166	**685**	**367**	**292**	**675**


*Novel alleles and novel sequence type (ST) are indicated in bold.*

Both *Ab* 6V and *Ab* 55 16S rRNA gene sequences show 100% identity with the type strain *Ab* RM4018 (Miller et al., 2007). ANI, AAI, and DDH analyses were performed with 22 strains within the *Arcobacter* group (Supplementary Table S1). *Campylobacter jejuni subsp. jejuni* NCTC 11168 and *Helicobacter pylori* 26695 were included as outgroups.

*Ab* 55 and *Ab* 6V have 98.06% nucleotide identity (Supplementary Table S2) and are comprised in the clustering including all the *A. butzleri* species. According to ANI, the closest relatives for both *Ab* 55 and *Ab 6*V are *Ab* NCTC 12481 (97.80% and 97.81 ANI, respectively) and *Ab* RM4018 (97.79% and 97.80 ANI). The same clustering is obtained by using AAI (Supplementary Table S3) with 97.60% between *Ab* 55 and *Ab* 6V, and 97.67 and 97.36% with *Ab* RM4018, respectively. DDH analysis confirmed the clustering obtained by ANI and AAI analysis, with values of 83.50 between *Ab* 6V and *Ab* 55, 81.00% between *Ab* 6V and *Ab* RM4018, and 81.30% between *Ab* 55 and *Ab* NCTC 12481 (Supplementary Table S4). As proposed by Chun et al. (2018) and, more specifically for *Arcobacter* spp., by On et al. (2017), these ANI and DDH values are within the range suggested to include *Ab* 55 and *Ab* 6V into the *A. butzleri* species. Moreover, our results support those achieved by Pérez-Cataluña et al. (2018a). Therefore, *Ab* 55 and *Ab* 6V should be placed within the *Aliarcobacter* gen. nov. as *Al. butzleri* comb. nov. (Pérez-Cataluña et al., 2018a,b), while for the definition of subspecies, a phenotypic comparison should support the *in silico* analyses (On et al., 2017).

### Protein Functional Classification

For *Ab* 55, 1173 UniProtKB AC/ID identifiers retrieved by PFAM annotator tools (Galaxy Tool Version 1.0.0) were successfully mapped to 1190 UniProtKB IDs (The UniProt Consortium, 2017). In *Ab* 55, the retrieved list included 33 genes associated with antibiotic resistance, including beta-lactamase, multidrug efflux pump, DNA gyrase, and resistance protein, and three with antibiotic biosynthesis related to bacteriocin, six associated with drug transmembrane transporter activity, 19 with virulence, four with hemolysis, and two with quorum sensing (*lux*S and *tqs*A).

For *Ab* 6V, 1189 out of 1189 UniProtKB AC/ID identifiers were mapped to 1207 UniProtKB. Based on their classification, we counted three genes associated with antibiotic biosynthesis, 32 related to antibiotic resistance, including beta-lactamase, multidrug efflux pump, DNA gyrase and resistance protein, six referred to drug transmembrane transporter activity, 22 associated with virulence, three with hemolysis, and two with quorum sensing (*lux*S and *tqs*A).

One of the few differences between these strains is the presence in *Ab* 6V of the hemolysin *tyl*C gene which codes for a protein with a conserved protein/cyclin M (CNNM) transmembrane domain, while, in *Ab* 55, we found only a precursor of hemolysin C. In both genomes, we identified the RNA methyltransferase hemolysin A (*tyl*A), which encodes for a 16S/23S rRNA (cytidine-2′-*O*)-methyltransferase that is considered a virulence factor in *H. pylori* infection and probably acts as a pore-forming toxin (Javadi and Katzenmeier, 2016).

**FIGURE 1:**
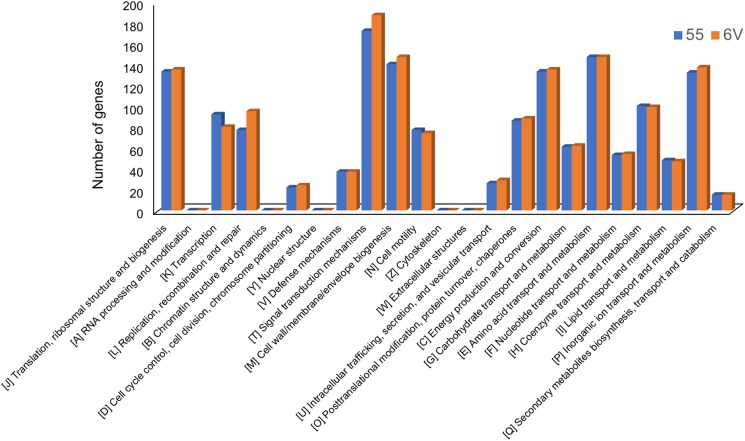
COGs functional classification of genes present in *A. butzleri* 55 and *A. butzleri* 6V genomes.

Overall, 1551 for *Ab* 55 and 1592 for *Ab* 6V predicted genes were assigned to the COG classification (Figure 1). The few differences in the distribution of genes into clusters of COG functional categories across *Ab* 55 and *Ab* 6V genomes, which emerged from Figure 1, supported the limited functional variability among these two strains, even though isolated from different samples and in different harvest seasons (Mottola et al., 2016).

Among COG categories, the cluster signal transduction mechanisms represented the largest group for both organism (172 genes, 11.09% for *Ab* 55 and 187 genes, 11.75% for *Ab* 6V), followed by amino acid transport and metabolism cluster (147 genes, 9.48% for *Ab* 55 and 147 genes, 9.23% for *Ab* 6V), Cell wall/membrane/envelope biogenesis cluster (140 genes, 9.03% for *Ab* 55 and 147 genes, 9.23% for *Ab* 6V) and energy production and conversion cluster (133 genes, 8.58% for *Ab* 55 and 135 genes, 8.48% for *Ab* 6V). These findings suggest that our *Arcobacter* strains have a versatile sensory transduction system and that for energy and carbon mainly rely on amino acid catabolism rather than on sugar fermentation, which is consistent with the ecological niches they have been isolated from.

### Virulence Determinants

The ability to adhere to various surfaces as well as chemotaxis, motility, and signal transduction play a pivotal role in the microbial survival and colonization of diverse ecological niches and can be involved in pathogenesis and antibiotic resistance (Richards et al., 2013; Chaban et al., 2015; Tiwari et al., 2017; Matilla and Krell, 2018).

**FIGURE 2:**
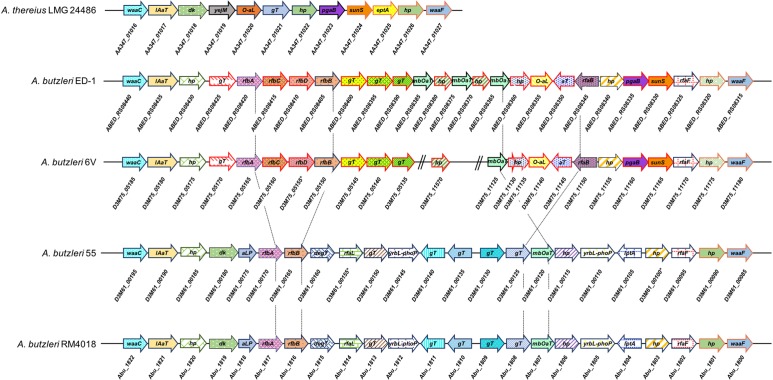
Genomic organization of waaC/waaF gene cluster in *A. thereius* LMG 24486, *A. butzleri* ED-1, *A. butlzeri* 6V, *A. butzleri* 55, and *A. butzleri* RM4018. Gene clustering is represented by the arrows superposed on the black horizontal line. Intergenic spaces are not drawn in scale. For *A. thereius* LMG 24486, *A. butzleri* ED-1, and *A. butzleri* RM4018, the locus tag of each gene is indicated below the respective gene arrow; for *A. butlzeri* 6V and *A. butzleri* 55, protein ID is indicated below the respective gene arrow. Red arrows in *A. butlzeri* ED-1 and *A. butzleri* 6V indicate genes with no orthologue in *A. butzleri* 55 and *A. butlzeri* RM4018. D3M75_05155 ^∗^ indicates pseudogene (frameshifted). lAaT: lipid A biosynthesis acylTransferase; dK: diacylglycerol kinase; yejM: inner membrane protein yejM; O-aL: O-antigen ligase; hP: hypotetical protein; gT: glycosyltransferase; pgaB: poly-beta-1,6-*N*-acetyl-D-glucosamine *N*-deacetylase; eptA: phosphoethanolamine transferase eptA; rfaF: lipopolysaccharide heptosyltransferase II; aT: acetyltransferase; sunS: glycosyltransferase sunS; aLP: alkaline phosphatase family protein; gPtT: glucose-1-phosphate thymidylyltransferase rfbA; yrbL-phoP: regulatory network protein; degT: DegT/DnrJ/EryC1/StrS family aminotransferase; rfbA: glucose-1-phosphate thymidyl transferase; rfbB: dTDP-glucose 4,6-dehydratase 1; rfbC: dTDP-4-dehydrorhamnose 3,5-epimerase; rfbD: dTDP-4-dehydrorhamnose reductase; mbOat: membrane bound *O*-acyl transferase; yrbL-phoP: YrbL-PhoP reg domain containing protein.

In both genomes, we identified orthologues of *waa*C and *waa*F genes, which are described as virulence determinant in *A. thereius* (Rovetto et al., 2017), but also in *Pseudomonas aeruginosa*, *E. coli*, *Klebsiella* *pneumonia*, and other *Campylobacteraceae* (Oldfield et al., 2002; DeLucia et al., 2011; Nilsson et al., 2018). waaF codes for a predicted ADP-heptose–LPS heptosyltransferase (*Ab* 55 D3M61_00085; *Ab* 6V D3M75_11180), involved in the biosynthesis of lipooligosaccharide (LOS); waaC codes for a lipopolysaccharide heptosyltransferase (*Ab* 55 D3M61_00195; *Ab* 6V D3M75_05185), which catalyzes the transfer of sugar moieties from activated donor molecules to specific acceptor molecules, forming glycosidic bonds. As in *Ab* RM4018 (Miller et al., 2007), which it shares the same content and organization with, in *Ab* 55, *waa*C and *waa*F genes are closely located suggesting the presence of a genetic cluster, whose composition is, however, different from *A. thereius* (Figure 2). In *Ab* 6V, the genetic locus comprised by *waa*C and *waa*F is similar to that of *Ab* ED-1 (26 genes) and contains several glycosyltransferases with no orthologues in *Ab* 55 and *Ab* RM4018. The outer genes of this locus are similar to *A. thereius*. However, in *Ab* 6V, these genes are located in three different contigs, which made only provisional, although likely, the reconstruction of the organization of the entire locus.

*Arcobacter butzleri*, as the other *Arcobacter* species, is motile by means of one single polar flagellum (Miller et al., 2007). The bacterial flagellar motor is comprised by a core structure (inner membrane stator complexes MotA4B2 and the C-ring), a dedicated type III secretion system (T3SS) export apparatus, the inner membrane MS-ring and the P- and L-rings. These core components are conserved across bacterial genera. Nevertheless, the architecture of flagellar motors in *A. butzleri* has a diverse (Rossmann and Beeby, 2018), “intermediate” (Chaban et al., 2018) motor structure. According to the analysis performed by Chaban et al. (2018), we retrieved homologous proteins of the flagellar system in *Ab* 55 and *Ab* 6V genomes (Table 3). In both strains, loci share the same genomic content and most of the flagellar genes are located within the same genomic locus. As reported by Chaban et al. (2018), the *Arcobacter*-type motor accessory proteins did not contain homologues of the accessory proteins FlgP, FlgQ, FlgT, while we identified the homologue FlgO, an outer membrane protein required for flagellar motility in *Vibrio cholerae*, highly conserved in *Vibrio* spp. (Zhu et al., 2017).

**Table 3:** Flagellum proteins in *Arcobacter butzleri* genomes.

		Organism	*A. butzleri* RM4018	*A. butzleri* 55	*A. butzleri* 6V
		Whole genome record	NC_009850.1	QXMK00000000	QXNB0000000
		Class	Epsilon	Epsilon	Epsilon
			GenBank accession	Protein ID	
T3SS	Flagellar biosynthesis protein FlhA	FlhA	ABV68160.1	D3M61_10075	D3M75_09250
	Flagellar biosynthetic protein FlhB	FlhB	ABV68164.1	D3M61_10055	D3M75_09230^∗^
	Flagellum-specific ATP synthase	FliI	ABV68162.1	D3M61_10065	D3M75_09240
	Flagellar biosynthetic protein FliP	FliP	ABV67255.1	D3M61_02925^∗^	D3M75_03525
	Flagellar biosynthetic protein FliQ	FliQ	ABV66480.1	D3M61_08725	D3M75_07160
	Flagellar biosynthesis protein FliR	FliR	ABV68165.1	D3M61_10050	D3M75_09225
C-ring	Flagellar motor switch protein FliG	FliG	ABV68184.1	D3M61_09955	D3M75_09130
	Flagellar motor switch protein FliM	FliM	ABV66477.1	D3M61_08740	D3M75_07145
	Flagellar motor switch protein FliN	FliN	ABV68182.1	D3M61_09965	D3M75_09140
MS-ring	Flagellar M-ring protein	FliF	ABV68185.1	D3M61_09950	D3M75_09125
Proximal rod	Flagellar hook-basal body protein FliE	FliE	ABV68168.1	D3M61_04895	D3M75_09210
	Flagellar basal body rod protein FlgB	FlgB	ABV68186.1	D3M61_09945	D3M75_09120
	Flagellar basal-body rod protein FlgC	FlgC	ABV68167.1	D3M61_10040	D3M75_09215
P-ring	Flagellar hook-associated protein	FlgI	A8ERA4.1	D3M61_08735	D3M75_07150
L-ring	Flagellar L-ring protein precursor	FlgH	ABV66485.1	D3M61_08700	D3M75_07185
Campy-specific	Flagellar lipoprotein	FlgP	n/a	n/a	n/a
	Hypothetical protein	FlgQ	n/a	n/a	n/a
	Tetratricopeptide repeat protein	PflA	WP_012147853.1	D3M61_10060	D3M75_09235
	Tetratricopeptide repeat protein	PflB	WP_012147861.1	D3M61_09990	D3M75_09165
Vibrio-specific	Flagellar assembly protein	FlgT	n/a	n/a	n/a
	Hypothetical protein	FlgO	WP_004511148.1	D3M61_08900	D3M75_07635


*^∗^Pseudogene (frameshifted).*

In *Ab* 55, the flagellar biosynthetic protein FliP (an integral membrane component) (Ferris and Minamino, 2006) was predicted as a pseudogene (frameshifted) (D3M61_02925), as the flagellar biosynthetic protein FlhB (a flagellar export component responsible for substrate specificity switching) (Minamino and Macnab, 2000) in *Ab* 6V (D3M75_09230). This might have occurred due to a homopolymeric region (AAAAAA) comprised in both the genomic loci which might have affected the base calling of the sequencing.

Both genomes harbor the genes *cj*1349 and *cad*F (coding for fibronectin-binding proteins CadF and Cj1349), *mvi*N (encoding a protein essential for the peptidoglycan biosynthesis) and *pld*A (encoding phospholipase A), the *gyr*A, *iro*E, and *irg*A (iron-regulating outer membrane protein), *cia*B (encoding the *C. jejuni* invasion antigen B) genes and the hemolysin gene *tyl*A, while only *Ab* 55 harbors *hecA* [a member of the filamentous hemagglutinin (FHA) family], and *hecB* (encoding a hemolysin activation protein), as occurs in the *Ab* RM4018 (Miller et al., 2007).

Cia proteins (including CiaB, CiaC, and CiaD) have been suggested to be involved in promoting internalization of *C. jejuni* for host invasion and require a full-length flagellar filament for proper secretion (Chaban et al., 2015).

Several authors have screened *A. butzleri* strains for the presence of virulence genes such as *cia*B, *cad*F, *cj*1349, *hec*A, and *irg*A, finding diverse prevalence of these genes in isolates from the same or different ecological niches (Collado et al., 2014; Ferreira et al., 2017; Rathlavath et al., 2017; Vicente-Martins et al., 2018). However, nucleotide sequence heterogeneity as well as PCR biases may provide false negative results thus underestimation of the actual prevalence of these genes. These drawbacks may be overcome by a genomic-based assessment, which, moreover, may allow the detection of novel (acquired) virulence genes.

Indeed, apart from the above-mentioned genes, recognized as putative virulence determinants in *A. butzleri* (Miller et al., 2007), we identified other genes coding for additional virulence associated protein in *Ab* 55 and *Ab* 6V genomes. Among these and present in *Ab* 55, *Ab* 6V, and *Ab* RM4018, we distinguished a DNA binding protein of the virulence factor B family, which contributes to the expression of virulence factors and to pathogenicity in *Staphylococcus aureus* (Matsumoto et al., 2010; Junecko et al., 2012), a VOC family virulence protein (glyoxalase/bleomycin resistance protein/dioxygenase superfamily domain), a conserved virulence factor B (DNA binding protein), and VirF of the AraC family of transcriptional regulators.

Only in *Ab* 55, with no orthologs in *Ab* 6V and *Ab* RM4018, we identified a virulence associated protein VirE (D3M61_07785), which has an 87% identity with a hypothetical protein of *Ab* L353 (WP_080952707.1); this was the only hit retrieved within *Arcobacter* genus (taxid: 28196) by BLASTP search in the NCBI database, while other results indicated identity of about 32% with a hypothetical protein of *Dyella* sp. 4G-K06 (WP_115495989.1) and with a primase from the *Escherichia* phage vB_EcoM-ep3. This sequence was also compared to metagenomic sequences comprised in the microbiome database MGNIFY (EMBL-EBI^©^; Mitchell et al., 2017) by BlastP analysis. Analysis showed 91% of identity with metagenomic sequence of a sample retrieved from Charlotte area wastewater treatment plants (North Carolina, United States). InterPro functional classification (Finn et al., 2017) assigned the protein the Virulence-associated E (IPR007936) family membership with a D-loop motif (Pfam:PF05272.5) and a related COG: 5545 Mobilome: prophage transposone category. The genomic locus in which the gene is located also comprises several hypothetical proteins, one Prophage CP4-57 integrase, several tRNA, one site-specific tyrosine recombinase XerC, putative DNA-invertase from lambdoid prophage Rac. The structure of this genomic locus is compatible with the presence of a genomic island (Juhas et al., 2009) and suggests the acquisition of this virulence element by a mechanism of horizontal gene transfer.

Furthermore, in *Ab* 6V genome, we also found one virulence sensor protein BvgS precursor and one virulence sensor histidine kinase PhoQ, which both have orthologues in *Ab* RM4018 (WP_012012740.1 and WP_012012731.1, respectively) but not in *Ab* 55.

Unique to *Ab* 6V, we identified two putative hemolysin activation/secretion proteins (D3M75_11460 and D3M75_03300), which have orthologues in *A. butzleri* ED-1, with a ShlB/FhaC/HecB family hemolysin secretion/ activation domain.

All together these findings lead to hypothesize a relatively high virulence of *A. butzleri* isolated from shellfish and prompt the need for a genomic analysis of more strains from this ecological niche, as well as for *in vitro* and *in vivo* studies, to unravel the mechanism of pathogenicity of this species.

### Antibiotic Susceptibility and Genetic Determinants

As shown in Table 4, both genomes harbor a wide endowment of genes involved in antibiotic resistance, including transporter, efflux pump, multidrug resistance protein and methyltransferase, although with some differences. As example, *Ab* 55 harbors a protein predicted as a bifunctional polymyxin resistance protein ArnA (D3M61_11465), which is not present neither in *Ab* 6V nor in the *A. butlzeri* RM4018. BLASTP analysis retrieved as best hit a hypothetical protein of *Campylobacter hyointestinalis* (WP_111949105.1).

**Table 4:** Antibiotic resistance genes in *Ab* 55 and *Ab* 6V genomes.

Gene name	Product	UniProt KB	Present in
*arnA*	Bifunctional polymyxin resistance protein ArnA	O52325	*Ab* 55
*arnB*	UDP-4-amino-4-deoxy-L-arabinose-oxoglutarate (polymyxin resistance)	Q8ZNF3	*Ab* 55 – *Ab* 6V
*bcr*	Bicyclomycin resistance protein	P28246	*Ab* 55 – *Ab* 6V
*bepD*	Efflux pump periplasmic linker BepD precursor	Q8G2M7	*Ab* 55 – *Ab* 6V
*bepE*	Efflux pump membrane transporter BepE	Q8G2M6	*Ab* 55 – *Ab* 6V
*bla*	Beta-lactamase OXA-15 precursor	Q51574	*Ab* 55 – *Ab* 6V
*cat3*	Chloramphenicol acetyltransferase 3	P00484	*Ab* 55 – *Ab* 6V
*eptA*	Phosphoethanolamine transferase EptA (polymyxin resistance)	P30845	*Ab* 55 – *Ab* 6V
*fsr*	Fosmidomycin resistance protein	P52067	*Ab* 55 – *Ab* 6V
*hcpC*	Putative beta-lactamase HcpC precursor	O25728	*Ab* 55 – *Ab* 6V
*hlpA*	Serine/threonine-protein kinase HipA (methicillin resistance)	P23874	*Ab* 6V
*ileS*	Isoleucine-tRNA ligase (mupirocine resistance)	P00956	*Ab* 55 – Ab6V
*lmrA*	Multidrug resistance ABC transporter ATP-binding and permease protein	Q9CHL8	*Ab* 6V
*-*	putative metallo-hydrolase (metallo Beta-lactamase)	Q5XD24	*Ab* 55
*macA*	Macrolide export protein MacA	P75830	*Ab* 55 – *Ab* 6V
*macB*	Macrolide export ATP-binding/permease protein MacB	P75831	*Ab* 55 – *Ab* 6V
*bla2*	Beta-lactamase 2 precursor	P10425	*Ab* 55 – *Ab* 6V
*mdtB*	Multidrug resistance protein MdtB	B7NCB1	*Ab* 55 – *Ab* 6V
*mdtE*	Multidrug resistance protein MdtE precursor	P37636	*Ab* 55
*mexA*	Multidrug resistance protein MexA	P52477	*Ab* 55 – *Ab* 6V
*mexB*	Multidrug resistance protein MexB	P52002	*Ab* 55 – *Ab* 6V
*oprM*	Outer membrane protein OprM	Q51487	*Ab* 55 – *Ab* 6V
*pbp*	Beta-lactam-inducible penicillin-binding protein	P07944	*Ab* 55 – *Ab* 6V
*relE*	mRNA interferase toxin RelE (ciprofloxacin and ampicillin)	P0C077	*Ab* 55 – *Ab* 6V
*rlmN*	Putative dual-specificity RNA methyltransferase RlmN (ribosome target antibiotics)	Q7A600	*Ab* 55 – *Ab* 6V
*sttH*	Streptothricin hydrolase	Q1MW86	*Ab* 55 – *Ab* 6V
*tetA*	Tetracycline resistance protein, class C	P02981	*Ab* 55 – *Ab* 6V
*uppP*	Undecaprenyl-diphosphatase (bacitracin resistance)	P60932	*Ab* 55 – *Ab* 6V
*wbpD*	Group B chloramphenicol acetyltransferase	G3XD01	*Ab* 55 – *Ab* 6V


The UDP-4-amino-4-deoxy-L-arabinose-oxoglutarate aminotransferase *arnB* gene was instead identified in both *Ab* 55 and *Ab* 6V, while it is not present in *A. butlzeri* RM4018. The coded protein belongs to the DegT/DnrJ/EryC1/StrS aminotransferase family protein, and it is required for resistance to polymyxin and cationic antimicrobial peptides (Lee and Sousa, 2014).

The predicted serine/threonine-protein kinase HipA belonging to the type II toxin-antitoxin system was only retrieved in *Ab* 6V predicted proteome (D3M75_03575), and it is involved in the methicillin resistance. Only in *Ab* 55, we identified the mRNA interferase toxin RelE (D3M61_04745), which is involved in ciprofloxacin and ampicillin resistance in *E. coli* (Harms et al., 2017).

Table 5 shows metal resistance genes annotated in *Ab* 55 and *Ab* 6V genomes.

**Table 5:** Metal resistance genes in *Ab* 55 and *Ab* 6V genomes.

Genes	Product	Metal resistance	Present in
*cadA*	Cadmium, zinc, and cobalt-transporting ATPase	Cadmium, zinc, cobalt	*Ab 6V – Ab 55*
*czcB*	Cobalt-zinc-cadmium resistance protein CzcB	Cadmium, zinc, cobalt	*Ab 6V*
*czcD*	Cadmium, cobalt and zinc/H(+)-K(+) antiporter	Cadmium, zinc, cobalt	*Ab 6V – Ab 55*
*copA_1*	Copper-exporting P-type ATPase A	Copper	*Ab 6V – Ab 55*
*copA_2*	Putative copper-importing P-type ATPase A	Copper	*Ab 6V – Ab 55*
*cusS*	Sensor kinase CusS	Copper	*Ab 6V – Ab 55*
Additional genes			
*arsC*	Arsenate reductase	Arsenic	*Ab 6V – Ab 55*
*modA*	Molybdate-binding periplasmic protein precursor	Molybdate chromate	*Ab 6V – Ab 55*
*copZ*	Copper chaperone CopZ	Copper	*Ab 6V – Ab 55*


Observational and experimental studies have highlighted that exposure of bacteria to heavy metals (mainly zinc and copper), mainly due to anthropogenic environmental contamination, can induce or co-select resistance to them and to one or more antibiotics. In particular, resistance may be induced by metals (i) via co-selection resistance, when different genes coding for antibiotic and metal resistance share a close location (as in mobile genetic elements, such as integrin, plasmid, or transponson), (ii) via cross-selection, when the same genetic element encodes for both antibiotic and metal resistance, and (iii) via co-regulation, when antibiotic and metal resistance genes share the same regulatory system (Lupo et al., 2012; Seiler and Berendonk, 2012; Chenia and Jacobs, 2017; Li et al., 2017; Poole, 2017; Yu et al., 2017; Wu et al., 2018). Nevertheless, as far as we know, only Otth et al. (2005) investigated susceptibility of *A. butzleri* to heavy metals, finding that all the 50 tested strains were susceptible to mercury, silver, and chrome salts, whereas all were resistant to molybdenum, manganese, nickel, cobalt, lead, and iron.

Metal resistance genes share the same location in both *Ab* 55 and *Ab* 6V genomes. *cad*A is close to genes coding for the ferrous iron transport protein FeoA and FeoB, outer membrane efflux proteins, transcriptional regulators, and several tRNA genes, suggesting the presence of a genomic island (Juhas et al., 2009). *czc*B is close to gene coding for the multidrug resistance protein MstB, one permease and the gene coding for the sensor protein ZraS. One *cop*A gene is close to the gene coding for outer membrane porin precursor and the copper sensing transcriptional repressor CsoR, while the other *cop*A is close to the ferrous iron transport proteins FeoA and FeoB. *cus*S is close to the transcriptional activator protein c*zc*R, *mac*B, and several chaperonine, whereas *ars*C is located near to genes coding for plasmid stabilization system protein and genes coding for flagellum biosynthesis (*fli*K and *flh*B), and cell division protein FtsA and FtsZ. *mod*A gene is located within a hypothetical operon including a transcriptional regulator GltR, a gene coding for a molybdenum-pterin binding protein, the regulator ModE, and the transport system permease protein ModB. *cop*Z is close to *mer*T gene, coding for a mercuric transport protein, a gene coding for a natural resistance-associated macrophage protein, a transcriptional regulatory protein ZraR and a sensor protein FixL.

The antimicrobial susceptibility of *Arcobacter* spp. isolated from various ecological niches has been investigated by several authors (Kabeya et al., 2004; Kayman et al., 2012; Ferreira et al., 2013, 2017; Scanlon et al., 2013; Collado et al., 2014; Rahimi, 2014; Yesilmen et al., 2014; Zacharow et al., 2015b; Aski et al., 2016; Van den Abeele et al., 2016; Elmali and Can, 2017; Rathlavath et al., 2017; Šilha et al., 2017; Soma et al., 2017; Vicente-Martins et al., 2018), but the lack of standardized protocols and interpretive criteria for the antimicrobial susceptibility testing (AST) of *Arcobacter* spp. is the major limitation for a comparable and univocal evaluation of antimicrobial resistance and susceptibility for these microorganisms (Ferreira et al., 2016). Results of the disk diffusion test are shown in Table 6, whereas in Table 7, are reported the MICs and MBCs assessed by broth microdilution method (section “Antimicrobial Susceptibility Testing”). The antimicrobial susceptibility pattern of *Ab* type strain LMG 10828^T^ (ATCC 49616, RM4018), used as a reference strain for the AST in our study, was comparable to that reported by Miller et al. (2007), for 10 out of 12 tested antibiotics, i.e., gentamicin, kanamycin, streptomycin, ciprofloxacin, chloramphenicol, erythromycin, vancomycin, ampicillin, cefotaxime, and penicillin G. Slight differences were related to susceptibility and resistance toward tetracycline and nalidixic acid, reported by Miller et al. (2007); in particular, in our test, the type strain resulted intermediate resistant toward both antibiotics. As concerns MIC values, the type strain *Ab* LMG10828 showed results consistent with those find by Riesenberg et al. (2017). Additionally, in our study, using a wider range of concentrations for penicillin and vancomycin, we were able to assess MIC values for *Ab* LMG10828^T^ toward these two antibiotics (i.e., 128 and 2048 μg/ml, respectively) that were previously reported by Riesenberg et al. (2017) as ≥64 μg/ml. According to MIC interpretive standards, *A. butzleri* LMG 10828^T^, *Ab* 55 and *Ab* 6V have been classified as resistant toward vancomycin and the three β-lactam antibiotics used in this study, i.e., cefotaxime (β-lactam cephalosporin), ampicillin, and penicillin G (β-lactam penicillins), confirming the results obtained by disk diffusion test (Tables 6, 7).

**Table 6:** Disk diffusion susceptibility test for *A. butzleri*.

		*A. butzleri* strain		
Class	Antibiotics	LMG 10828	55	6V
β-Lactams	Ampicillin 10 μg/disk	R^∗^	R^∗^	R^∗^
	Penicillin G 10 units/disk	R^∗^	R^∗^	R^∗^
	Cefotaxime 30 μg/disk	R^∗^	R^∗^	R^∗^
Glycopeptides	Vancomycin 30 μg/disk	R^∗^	R^∗^	R^∗^
Quinolones	Ciprofloxacin 5 μg/disk	S	S	S
	Nalidixic acid 30 μg/disk	I	R	I
Aminoglycosides	Gentamicin 10 μg/disk	S	S	S
	Kanamycin 30 μg/disk	S	S	S
	Streptomycin 10 μg/disk	S	S	S
Phenicol	Chloramphenicol 30 μg/disk	R	I	I
Tetracycline	Tetracycline 30 μg/disk	I	R	R
Macrolide	Erytromycin 15 μg/disk	I	R	R


*Classification as S (susceptible), I (intermediate), and R (resistant) was carried out according to zone diameter interpretive standards for *Staphylococcus* spp. (erytromycin and penicillin G) and *Enterobacteriaceae* (ampicillin, gentamicin, cefotaxime, ciprofloxacin, tetracycline, chloramphenicol, nalidixic acid, kanamycin, and streptomycin) (CLSI, 2015; Elmali and Can, 2017). To our knowledge, no vancomycin reference interpretive criteria are reported for *A. butzleri*. ^∗^No inhibition zone detected.*

**Table 7:** Cefotaxime, ampicillin, penicillin G, and vancomycin MIC and MBC values for *A. butzleri.*

	β-Lactams						Glycopeptide	
*A. butzleri* strain	Cefotaxime μg/ml		Ampicillin μg/ml		Penicillin G μg/ml		Vancomycin μg/ml	
	MIC^a^	MBC^b^	MIC^a^	MBC^b^	MIC^a^	MBC^b^	MIC^a^	MBC^b^

LMG 10828	16	16	32	32	128	256	2,048	>2,048
55	128	128	256	256	1,024	1,024	>2,048	>2,048
6V	64	64	64	64	256	256	2,048	>2,048


*^*a*^Minimal inhibitory concentration. ^*b*^Minimal bactericidal concentration.*

For *A. butzleri* type strain, *Ab* 55 and *Ab* 6V, the highest MIC and MBC values were observed for vancomycin, which were ≥2048 μg/ml (Table 7).

As recently reviewed by Ahmed and Baptiste (2018) in enterococci, molecular basis for vancomycin resistance phenotypes are determined by the presence of the vancomycin resistance (Van) operons (described as vanA, -B, -C, -D, -E, -G, -L, -M, and N) which may be located on the chromosome or on a plasmid. No element of the described operons was found in *Ab* 55 and *Ab* 6V genomes, as well as in *Ab* RM4018. However, the resistance of Gram-negative bacteria toward vancomycin may be intrinsic due to the inability of glycopeptides [molecules with high molecular weight (1450–1500 Da) and size] to pass through porins, which govern the movement of hydrophilic molecules across their outer membrane (Quintiliani and Courvalin, 1995) to reach their site of action, i.e., the cell wall (Nicolosi et al., 2010). Indeed, 100% of the *A. butzleri* isolates tested by Aski et al. (2016), Rathlavath et al. (2017), and Soma et al. (2017) were resistant to vancomicyn.

Both strains are resistant to all the ß-lactam antibiotics tested (ampicillin, penicillin, and cefotaxime) (Table 6). The resistance of *A. buzleri* isolates to β-lactams is widespread in seafood and water sources (Collado et al., 2014; Rathlavath et al., 2017; Šilha et al., 2017) as well as in other environments (Kayman et al., 2012; Ferreira et al., 2013; Rahimi, 2014; Yesilmen et al., 2014; Zacharow et al., 2015b; Aski et al., 2016; Van den Abeele et al., 2016; Elmali and Can, 2017; Vicente-Martins et al., 2018). Rathlavath et al. (2017) found that on 40 *A. butzleri* isolated from shellfish, 100% resulted resistant to cefotaxime and 70% to ampicillin, while among 81 *A. butzleri* isolated from fish, 98.7% was resistant to cefotaxime and 72.8% to ampicillin. Moreover, of 26 *A. butzleri* isolated from coastal water, 100% was resistant to cefotaxime and 73% to ampicillin (Rathlavath et al., 2017). Šilha et al. (2017) found that 94.4% of 18 *A. butzleri* isolated from water sources were resistant to ampicillin and 100% were resistant to penicillin G, while Collado et al. (2014) found that 45.2% of 62 *A. butzleri* isolated from bivalve molluscs were resistant to ampicillin.

The ß-lactam resistance is generally due to the combined effects of the presence and activity of ß-lactamase genes, of the binding to targets (penicillin-binding proteins) and, in Gram-negative bacteria, of the outer-membrane permeability (Georgopapadakou, 1993). In the genomes of *Ab* 55 and *Ab* 6V, we identified three putative ß-lactamases orthologues to that of *A. butlzeri* RM4018 (Miller et al., 2007) [MBL fold metallo-hydrolase D3M61_00510 and D3M61_04375 (*Ab* 55), D3M75_05505 and D3M75_08430(*Ab* 6V); class D beta-lactamase D3M61_10735, D3M75_10375 (*Ab* 6V)] as well as penicillin binding proteins (*Ab* 6V D3M75_03520, D3M75_10675, D3M75_10720; *Ab* 55: D3M61_02930, D3M61_10940, D3M61_10985).

Furthermore, in the genomes of both strains, we retrieved the lrg*AB* operon, which modulates penicillin tolerance in *Staphylococcus* (Bayles, 2000; Groicher et al., 2000) and was previously suggested as the ß-lactam resistance enhancer in *A. butzleri* RM4018 (Miller et al., 2007).

**FIGURE 3:**
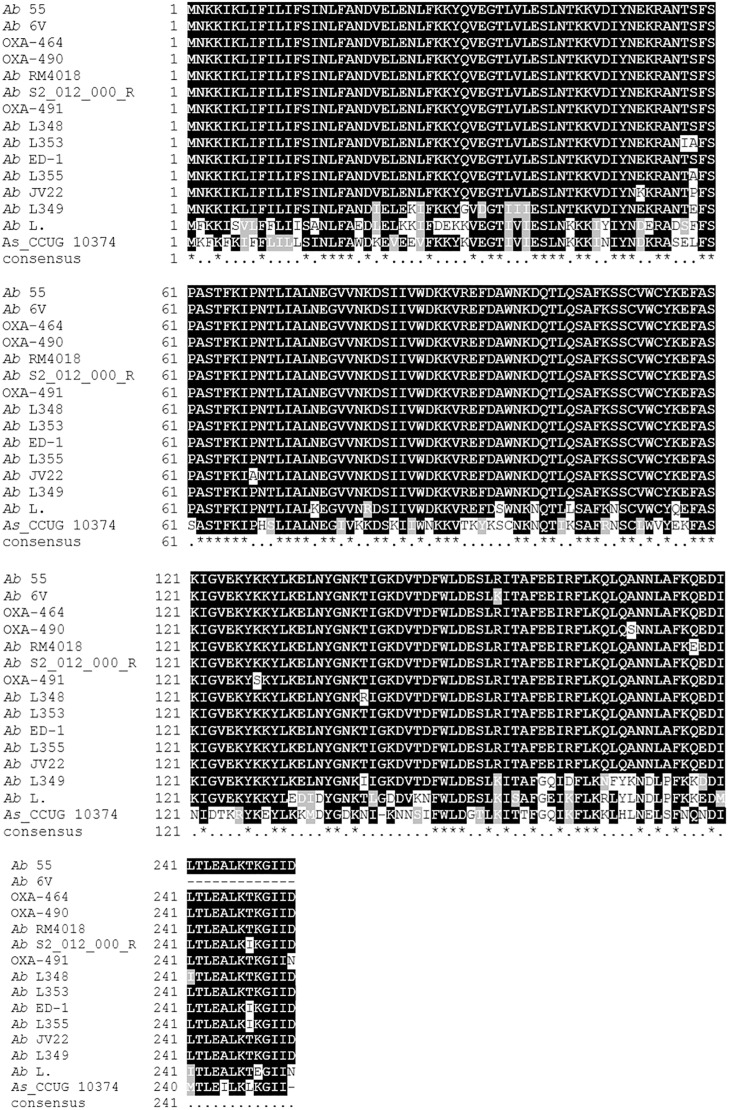
Multialignment of OXA beta-lactamase from *Arcobacter butzleri* obtained by using T-Coffee web server (Di Tommaso et al., 2011). *Ab* 55 protein ID: D3M61_10735; Ab 6V: D3M75_10375; OXA-491, Accession: ANW35665.1; OXA-464: ANW35663.1; OXA-490: ANW35664.1. *A. butzleri* RM4018: WP_012013127.1; *A. butzleri* S2_012_000_R2_80: PZP12670.1; *A. butzleri* L348: WP_046997374.1; *A. butzleri* L353: WP_050071304.1; *A. butzleri* ED-1: WP_014468976.1; *A. buzleri* L355: WP_046997672.1; *A. butzleri* JV22: EFU68937.1; *A. butzleri* L349: WP_046993700.1; *A. butzleri* L.: WP_014474670.1; *A. skirrowii* CCUG 10374: WP_115588490.1.

Figure 3 shows the multialignment of beta-lactamase protein in *Ab* 55 and *Ab* 6V and other *Arcobacter* species. In *Ab* 6V the predicted protein (D3M75_10375) is truncated at N-terminal due to a nucleotide mutation in the genomic locus which leads to a premature stop codon. The DNA sequence translated by EMBOSS^®^ Sixpack shows that changing the reading frame would recover the entire protein, identical to *Ab* 55 D3M61_10735. The sequence of *Ab* 55 D3M61_10735 share 100% of identity with OXA-464, which differs with the ß-lactamase of the type strain *A. butleri* RM4018 for a glutamine instead of glutamic acid in position 177.

*Ab* 55 and *Ab* 6V as well as the *Ab* type strain LMG 10828 (RM4018) were sensitive to the three aminoglycoside antibiotics used in this study namely gentamicin, kanamycin, and streptomycin (Table 6). Our results are in agreement with those obtained by Rathlavath et al. (2017) on the 147 *A. butzleri* isolates from seafood (40 isolates were from shellfish) and coastal water, which were all sensitive to these three antibiotics. Susceptibility to these antibiotics is also consistent to that found by other authors who reported susceptibility to streptomycin and/or gentamicin and/or kanamycin for the great majority (96.4–100%) of *A. butzleri* isolated from shellfish [gentamicin (Collado et al., 2014)], water sources [gentamicin and streptomycin (Šilha et al., 2017)] and other various sources [gentamicin (Kayman et al., 2012; Ferreira et al., 2013, 2017; Van den Abeele et al., 2016); gentamicin and streptomycin (Šilha et al., 2017); kanamycin and streptomycin (Kabeya et al., 2004); gentamicin, kanamycin and streptomycin (Rahimi, 2014)]. Aminoglycosides are proposed as antibiotics to be used in *Arcobacter* infections (Rahimi, 2014; Ferreira et al., 2016; Rathlavath et al., 2017). Nevertheless, a study reported high percentage (80%) of resistance to gentamicin and kanamycin in *A. butzleri* isolates from porcine samples (Scanlon et al., 2013) but only five isolates were tested. Mechanisms of bacterial resistance to this class of antibiotics are diverse including the inactivation by aminoglycoside modifying enzymes, mutation of the ribosome target, and modification of the ribosome by methyltransferase enzymes (Wilson, 2014; Garneau-Tsodikova and Labby, 2016). Resistance may also arise from mutation in the rrs encoding for 16S rRNA, even if these mutations are quite rare, as they would interfere with the vital cellular machinery.

Susceptibility to the hydrophilic fluoroquinolone ciprofloxacin or very low percentage of resistant isolates (0–3.2%) are widely reported in literature (Kayman et al., 2012; Collado et al., 2014; Rahimi, 2014; Ferreira et al., 2017; Rathlavath et al., 2017; Šilha et al., 2017; Soma et al., 2017), although some authors reported percentages ranging from 12.7 to 55.8% of *A. butzleri* resistant to ciprofloxacin isolated from patients with gastroenteritis, retail food products, and poultry slaughterhouse (Ferreira et al., 2013; Zacharow et al., 2015b; Van den Abeele et al., 2016; Vicente-Martins et al., 2018). The acquisition of fluoroquinolone resistance may represent a serious issue for the health care system since they are the first-choice antibiotic for treating *Campylobacter* infection in humans and they were suggested to be used in *Arcobacter* enteritis (Vandenberg et al., 2006; Collado et al., 2014; Ferreira et al., 2016).

Our strains are susceptible to ciprofloxacin. Indeed, we did not find in *A. butzleri* *Ab* 55 and *Ab* 6V the mutation in the quinolone resistance determining region in position 254 of the *gyr*A gene which causes a transition from cytosine to thymine leading to the substitution of a threonine to isoleucine (Abdelbaqi et al., 2007).

*Ab* LMG10828^T^ (RM 4018) and *Ab* 6V were intermediate resistant to the hydrophobic quinolone nalidixic acid, whereas *Ab* 55 was resistant to this antibiotic. However, the absence of *gyr*A mutations in these strains suggests that the putative resistance could be due to the mechanisms of hydrophobic quinolones uptake as suggested by Miller et al. (2007). High percentage of resistance toward the nalidixic acid was reported for 77.5 and 83.4% of *A. butzleri* isolated from shellfish by Rathlavath et al. (2017) and Collado et al. (2014), respectively, for 71.6% of *A. butzleri* isolates from fish (Rathlavath et al., 2017) and for 57.6 and 88.9% of *A. butzleri* isolates from water sources in two different studies (Rathlavath et al., 2017; Šilha et al., 2017). One hundred percent of *A. butzleri* isolated from other various sources, namely, retail food products, porcine, slaughterhouse, and dairy plant samples, by different authors, were resistant to nalidixic acid (Scanlon et al., 2013; Elmali and Can, 2017; Ferreira et al., 2017; Vicente-Martins et al., 2018). By contrast, high percentage of susceptibility toward nalidixic acid, ranging from 50 to 77.8%, was reported by Yesilmen et al. (2014) and Kayman et al. (2012) for *A. butzleri* isolated from milk and dairy products and human gastroenteritis stool samples, respectively. However, no more than 10 *A. butzleri* isolates were tested in both studies.

*Arcobacter butzleri* *Ab* 55 and *Ab* 6V are intermediate resistant to chloramphenicol (Table 6). Differences about susceptibility against this antibiotic were reported among several studies (Ferreira et al., 2016); e.g., Rathlavath et al. (2017) reported high susceptibility rates ranging from 65.3 to 77.7% for 147 *A. butzleri* isolates from shellfish, fish, and coastal water whereas Šilha et al. (2017) reported that 66.6% of *A. butzleri* isolated from water sources was resistant. Also for *A. butzleri* isolated from various sources (poultry meat and other retail food products, poultry slaughterhouse, and animal and human stool samples) variable resistance percentages were reported ranging from 2.3 to 87.7% (Ferreira et al., 2013; Rahimi, 2014; Aski et al., 2016; Šilha et al., 2017; Soma et al., 2017; Vicente-Martins et al., 2018). Otth et al. (2004) suggested that it may depend on local differences in the usage of this antibiotic.

Chloramphenicol resistance commonly consists of its enzymatic inactivation mainly by acetyltransferases or occasionally by phosphotransferases, but additional mechanisms involve the presence of efflux pump which act as extrusion transporters, mutation or modification of the target site, and decreased outer membrane permeability. In the genomes of *Ab* 55 and *Ab* 6V, we retrieved the *cat*3 gene encoding for a type A chloramphenicol *O*-acetyltransferase (*Ab* 55 D3M61_09345; *Ab* 6V D3M75_06440), which catalyzes the acetyl-CoA-dependent acetylation of chloramphenicol and they resulted as intermediate resistant.

Our *A. butzleri* strains *Ab* 55 and *Ab* 6V are resistant to tetracycline (Table 6). As for chloramphenicol, tetracycline susceptibility results vary among studies, even if tetracycline is proposed for treating *Arcobacter* infections by different authors (Ferreira et al., 2016; Rathlavath et al., 2017). Rathlavath et al. (2017) and Šilha et al. (2017) reported that 100 and 77.8% of *A. butzleri* isolated from shellfish, fish, coastal water, and water sources, respectively, were susceptible to tetracycline and high susceptibility rates, ranging from 78.2 to 100% were reported also for *A. butzleri* isolated from poultry meat, human and animal stool samples (Rahimi, 2014; Aski et al., 2016; Šilha et al., 2017). Conversely Vicente-Martins et al. (2018) reported that 95.4% of 65 *A. butzleri* strains isolated from retail food products was resistant to tetracycline and also Yesilmen et al. (2014) reported that 100% of *A. butzleri* isolated from milk and cheese was resistant toward this antibiotic, even if only 10 isolates were tested.

Tetracycline resistance may be due to several mechanisms: efflux, modification, protection from the ribosome binding, modification of 16S rRNA at the tetracycline binding site. These mechanisms are mediated by different proteins, among which Tet(O) and Tet(M) are the most important. These proteins are paralogues of the translational GTPase EF-G which removes tetracycline from its inhibitory site on the ribosome through a GTP-dependent hydrolysis. Both are part of a larger group of proteins called ribosomal protection proteins (RPPs), which also includes Tet(Q), Tet(S), Tet(T), Tet(W), and OtrA (Chopra and Roberts, 2001). In the genomes of our strains, we retrieved proteins predicted as tetracycline resistance protein of class C (MFS transporter-multidrug efflux pump) with 27% identity with the orthologues in *E. coli* and elongation factors with the same domain found in the C terminus of RPPs Tet(M) and Tet(O), with 65% identity with EF-G of *E. coli*.

Concerning erythromycin, *A. butzleri* *Ab* 55 and *Ab* 6V are resistant to this antibioitc (Table 6). However, different percentages of erythromycin resistance were reported in several studies. In particular, *A. butzleri* isolated from seafood and water sources, as well as from poultry meat, animal and human stool samples, dairy plant, and cheese were found to be susceptible to this antibiotic at percentages ranging from 65 to 100% (Kayman et al., 2012; Collado et al., 2014; Aski et al., 2016; Ferreira et al., 2017; Rathlavath et al., 2017; Šilha et al., 2017). Zacharow et al. (2015b) and Soma et al. (2017) reported that 62 and 50%, respectively, of *A. butzleri* isolated from animals, humans, and foods of animal origin were resistant toward this antibiotic whereas Yesilmen et al. (2014) and Scanlon et al. (2013) reported that 80% of *A. butzleri* isolated from milk, cheese, and porcine samples, respectively, were resistant too, but no more than 10 isolates were tested.

Erythromycin resistance is due, besides the less common mutation in 23S rRNA or ribosomal proteins, to post-transcriptional methylation of an adenine residue in 23S caused by the action of erm class gene-coded methylases in Gram-positive bacteria (Kurincic et al., 2007). Ribosomal RNA small subunit methyltransferases were also present in *Ab* 55 (D3M61_05905) and *Ab* 6V (D3M75_01835) genomes with 26 and 27% identity with reference sequence of *Enterococcus faecium* (Accession: YP_004172630.1), respectively; this protein is also present in the genome of *A. butzleri* RM4018, although it results intermediate resistant in response to erythromycin. Furthermore, we identified *mac*A and *mac*B genes encoding for macrolide exporter proteins in both *Ab* 55 and *Ab* 6V. The single copy of 23S rRNA of both *Ab* 55 and *Ab* 6V does not present any of the identified mutation responsible for erythromycin resistance.

## Conclusion

Genomic analyses herein performed allowed us to confirm the recently (Pérez-Cataluña et al., 2018a,b) suggested amendment of *A. butzleri* as *Al. butzlerii*, comb. nov.

Antimicrobial susceptibility tests defined *Ab* 55 and *Ab* 6V strains as resistant to vancomycin, tetracyclin, nalidixic acid (only *Ab* 55 whereas *Ab* 6V is intermediate resistant), erythromycin, and β-lactam antibiotics. Moreover, in our strains isolated from shellfish, we identified numerous virulence, antibiotic, and heavy metal resistance determinants, also additional to those previously found in the genome sequenced *A. butzleri* ED-1, isolated from fuel cell, and in the *A. butzleri* type strain RM 4018, isolated from human gastroenteritis (Miller et al., 2007; Pérez-Cataluña et al., 2018a), leading to hypothesize that shellfish strain may be potentially more virulent.

The findings of food-related *A. butzleri* support both epidemiological surveillance and food safety risk assessment and management in the shellfish industry.

Further analysis in our laboratories is ongoing to sequence and characterize other *A. butleri* strains isolated from shellfish and from other food matrices, in order to obtain an updated description of the species and to clarify the role of genetic endowment, as well as of the ecological niches the strain come from, in the pathogenesis of *A. butzleri.* The genomic sequences here presented, and the novel insights obtained in the present study appreciably contribute to achieve these goals.

## Author Contributions

VF conceived the work, interpreted the data, and organized the bioinformatic work. FF performed the genomic sequencing and the bioinformatic work. DC and FB performed the antimicrobial susceptibility testing. VF and FF wrote the manuscript. All the authors contributed to the revision of the manuscript and read and approved the submitted manuscript.

## Conflict of Interest Statement

The authors declare that the research was conducted in the absence of any commercial or financial relationships that could be construed as a potential conflict of interest.

## Footnotes

1 https://pubmlst.org/arcobacter

2 www.ispa.cnr.it/Collection

3 https://www.ncbi.nlm.nih.gov/COG/

4 http://blast.ncbi.nlm.nih.gov/Blast.cgi

5 http://bccm.belspo.be

## References

[B1] Abdelbaqi K. Ménard A. Prouzet-Mauleon V. Bringaud F. Lehours P. Mégraud F. (2007). Nucleotide sequence of the *gyrA* gene of *Arcobacter* species and characterization of human ciprofloxacin-resistant clinical isolates. *FEMS Immunol. Med. Microbiol.* 49 337–345. 10.1111/j.1574-695X.2006.00208.x 17378897

[B2] Afgan E. Baker D. van den Beek M. Blankenberg D. Bouvier D. Čech M. (2016). The Galaxy platform for accessible, reproducible and collaborative biomedical analyses: 2016 update. *Nucleic Acids Res.* 44 W3–W10. 10.1093/nar/gkw343 27137889 PMC4987906

[B3] Ahmed M. O. Baptiste K. E. (2018). Vancomycin-Resistant *Enterococci*: a review of antimicrobial resistance mechanisms and perspectives of human and animal health. *Microbiol. Drug Resist.* 24 590–606. 10.1089/mdr.2017.0147 29058560

[B4] Arguello E. Otto C. C. Mead P. Babady N. E. (2015). Bacteremia caused by *Arcobacter butzleri* in an immunocompromised host. Carroll KC, ed. *J. Clinic. Microbiol.* 53 1448–1451. 10.1128/JCM.03450-14 25673792 PMC4365236

[B5] Aski H. S. Tabatabaei M. Khoshbakht R. Raeisi M. (2016). Occurrence and antimicrobial resistance of emergent *Arcobacter* spp. isolated from cattle and sheep in Iran. *Comp. Immunol. Microbiol. Infect. Dis.* 44 37–40. 10.1016/j.cimid.2015.12.002 26851593

[B6] Auch A. F. von Jan M. Klenk H. P. Göker M. (2010). Digital DNA-DNA hybridization for microbial species delineation by means of genome-to-genome sequence comparison. *Stand. Genomic Sci.* 2 117–134. 10.4056/sigs.531120 21304684 PMC3035253

[B7] Aziz R. K. Bartels D. Best A. A. DeJongh M. Disz T. Edwards R. A. (2008). The RAST Server: rapid annotations using subsystems technology. *BMC Genomics* 8:75. 10.1186/1471-2164-9-75 18261238 PMC2265698

[B8] Banting G. Figueras Salvat M. J. (2017). “*Arcobacter*,” in *Global Water Pathogens Project*, eds Rose J. B. Jiménez-Cisneros B. (East Lansing, MI: Michigan State University)

[B9] Bayles K. W. (2000). The bactericidal action of penicillin: new clues to an unsolved mystery. *Trends Microbiol.* 8 274–278. 10.1016/S0966-842X(00)01762-5 10838585

[B10] Chaban B. Coleman I. Beeby M. (2018). Evolution of higher torque in *Campylobacter*-type bacterial flagellar motors. *Sci. Rep.* 8:97. 10.1038/s41598-017-18115-1 29311627 PMC5758724

[B11] Chaban B. Hughes H. V. Beeby M. (2015). The flagellum in bacterial pathogens: for motility and a whole lot more. *Semin. Cell Dev. Biol.* 46 91–103. 10.1016/j.semcdb.2015.10.032 26541483

[B12] Chenia H. Y. Jacobs A. (2017). Antimicrobial resistance, heavy metal resistance and integron content in bacteria isolated from a South African tilapia aquaculture system. *Dis. Aquat. Organ* 126 199–209. 10.3354/dao03173 29160218

[B13] Chopra I. Roberts M. (2001). Tetracycline antibiotics: mode of action, applications, molecular biology, and epidemiology of bacterial resistance. *Microbiol. Mol. Biol. Rev.* 65 232–260. 10.1128/MMBR.65.2.232-260.2001 11381101 PMC99026

[B14] Chun J. Oren A. Ventosa A. Christensen H. Arahal D. R. da Costa M. S. (2018). Proposed minimal standards for the use of genome data for the taxonomy of prokaryotes. *Int. J. Syst. Evol. Microbiol.* 68 461–466. 10.1099/ijsem.0.002516 29292687

[B15] CLSI (2015). *Performance Standards for Antimicrobial Susceptibility Testing; Twenty-Fifth Informational Supplement. CLSI document M100-S25.* Wayne, PA: Clinical and Laboratory Standards Institute.

[B16] Collado L. Figueras M. J. (2011). Taxonomy, epidemiology, and clinical relevance of the genus *Arcobacter*. *Clin. Microb. Rev.* 24 174–192. 10.1128/CMR.00034-10 21233511 PMC3021208

[B17] Collado L. Jara R. Vásquez N. Telsaint C. (2014). Antimicrobial resistance and virulence genes of *Arcobacter* isolates recovered from edible bivalve molluscs. *Food Control.* 46 508–512. 10.1016/j.foodcont.2014.06.013

[B18] DeLucia A. M. Six D. A. Caughlan R. E. Gee P. Hunt I. Lam J. S. (2011). Lipopolysaccharide (LPS) inner-core phosphates are required for complete LPS synthesis and transport to the outer membrane in *Pseudomonas aeruginosa* PAO1. *mBio* 2:e00142-11. 10.1128/mBio.00142-11 21810964 PMC3147165

[B19] Di Tommaso P. Moretti S. Xenarios I. Orobitg M. Montanyola A. Chang J. M. (2011). T-Coffee: a web server for the multiple sequence alignment of protein and RNA sequences using structural information and homology extension. *Nucleic Acids Res.* 39 W13–W17. 10.1093/nar/gkr245 21558174 PMC3125728

[B20] Douidah L. De Zutter L. Van Nieuwerburgh F. Deforce D. Ingmer H. (2014). Presence and analysis of plasmids in human and animal associated *Arcobacter* species. *PLoS One* 9:e85487. 10.1371/journal.pone.0085487 24465575 PMC3896396

[B21] Douidah L. de Zutter L. Baré J. De Vos P. Vandamme P. Vandenberg O. (2012). Occurrence of putative virulence genes in *Arcobacter* species isolated from humans and animals. *J. Clin. Microbiol.* 50 735–741. 10.1128/JCM.05872-11 22170914 PMC3295157

[B22] Elmali M. Can H. Y. (2017). Occurrence and antimicrobial resistance of *Arcobacter* species in food and slaughterhouse samples. *Food Sci. Technol.* 37 280–285. 10.1590/1678-457x.19516

[B23] Engberg J. On S. L. Harrington C. S. Gerner-Smidt P. (2000). Prevalence of *Campylobacter*, *Arcobacter*, *Helicobacter* and *Sutterella* spp. in human fecal samples as estimated by a reevaluation of isolation methods for Campylobacters. *J. Clin. Microbiol.* 38 286–291. 10618103 10.1128/jcm.38.1.286-291.2000 PMC88711

[B24] Ercolini D. Fusco V. Blaiotta G. Sarghini F. Coppola S. (2005). Response of *Escherichia coli* O157:H7, *Salmonella Thyphimurium*, *Listeria monocytogenes* and *Staphylococcus aureus* to the stresses occurring in model manufactures of Grana Padano cheese. *J. Dairy Sci.* 88 3818–3825. 10.3168/jds.S0022-0302(05)73067-8 16230687

[B25] Ferreira S. Fraqueza M. J. Queiroz J. A. Domingues F. C. Oleastro M. (2013). Genetic diversity, antibiotic resistance and biofilm-forming ability of *Arcobacter butzleri* isolated from poultry and environment from a Portuguese slaughterhouse. *Int. J. Food Microbiol.* 162 82–88. 10.1016/j.ijfoodmicro.2013.01.003 23369730

[B26] Ferreira S. Júlio C. Queiroz J. A. Domingues F. C. Oleastro M. (2014a). Molecular diagnosis of *Arcobacter* and *Campylobacter* in diarrhoeal samples among Portuguese patients. *Diagn. Microbiol. Infect. Dis.* 78 220–225. 10.1016/j.diagmicrobio.2013.11.021 24361090

[B27] Ferreira S. Oleastro M. Domingues F. C. (2017). Occurrence, genetic diversity and antibiotic resistance of *Arcobacter* sp. in a dairy plant. *J. Appl. Microbiol.* 123 1019–1026. 10.1111/jam.13538 28712149

[B28] Ferreira S. Queiroz J. A. Oleastro M. Domingues F. C. (2014b). Genotypic and phenotypic features of *Arcobacter butzleri* pathogenicity. *Microb. Pathog.* 76 19–25. 10.1016/j.micpath.2014.09.004 25218724

[B29] Ferreira S. Queiroz J. A. Oleastro M. Domingues F. C. (2016). Insights in the pathogenesis and resistance of *Arcobacter*: a review. *Crit. Rev. Microbiol.* 42 364–383. 10.3109/1040841X.2014.954523 25806423

[B30] Ferris H. U. Minamino T. (2006). Flipping the switch: bringing order to flagellar assembly. *Trends Microbiol.* 14 519–526. 10.1016/j.tim.2006.10.006 17067800

[B31] Figueras M. J. Collado L. Levican A. Perez J. Solsona M. J. Yustes C. (2011a). Arcobacter molluscorum sp. nov., a new species isolated from shellfish. *Syst. Appl. Microbiol.* 34 105–109. 10.1016/j.syapm.2010.10.001 21185143

[B32] Figueras M. J. Levican A. Collado L. Inza M. I. Yustes C. (2011b). *Arcobacter ellisii* sp. nov., isolated from mussels. *Syst. Appl. Microbiol.* 34 414–418. 10.1016/j.syapm.2011.04.004 21723060

[B33] Figueras M. J. Levican A. Pujol I. Ballester F. Rabada Quilez M. J. Gomez-Bertomeu F. (2014). A severe case of persistent diarrhoea associated with *Arcobacter cryaerophilus* but attributed to *Campylobacter* sp. and a review of the clinical incidence of *Arcobacter* spp. *New Microbes New Infect.* 2 31–37. 10.1002/2052-2975.35 25356338 PMC4184587

[B34] Finn R. D. Attwood T. K. Babbitt P. C. Bateman A. Bork P. Bridge A. J. (2017). InterPro in 2017 - beyond protein family and domain annotations. *Nucleic Acids Res.* 45 D190–D199. 10.1093/nar/gkw1107 27899635 PMC5210578

[B35] Flynn K. Villarreal B. P. Barranco A. Belc N. Bjornsdottir B. Fusco V. (2019). An introduction to current food safety needs. *Trends Food Sci. Technol.* 84 1–3. 10.1016/j.tifs.2018.09.012

[B36] Fong T. T. Mansfield L. S. Wilson D. L. Schwab D. J. Molloy S. L. Rose J. B. (2007). Massive microbiological groundwater contamination associated with a waterborne outbreak in Lake Erie, South Bass Island, Ohio. *Environ. Health Perspect.* 115 856–864. 10.1289/ehp.9430 17589591 PMC1892145

[B37] Franz C. M. A. P. den Besten H. M. W. Böhnlein C. Gareis M. Zwietering M. H. Fusco V. (2018). Microbial food safety in the 21st century: emerging challenges and foodborne pathogenic bacteria. *Trends Food Sci. Technol.* 81 155–158. 10.1016/j.tifs.2018.09.019

[B38] Fusco V. Abriouel H. Benomar N. Kabisch J. Chieffi D. Cho G.-S. (2018). “Opportunistic foodborne pathogens,” in *Food Safety and Preservation: Modern Biological Approaches to Improving Consumer Health*, 1st Edn, Chap. 10 eds Grumezescu A. Holban A. M. (Academic Press), 269–306. 10.1016/B978-0-12-814956-0.00010-X

[B39] Fusco V. Quero G. M. Morea M. Blaiotta G. Visconti A. (2011). Rapid and reliable identification of *Staphylococcus aureus* harbouring the enterotoxin gene cluster (*egc*) and quantitative detection in raw milk by real time PCR. I. *J. Food Microbiol.* 144 528–537. 10.1016/j.ijfoodmicro.2010.11.016 21131084

[B40] Garneau-Tsodikova S. Labby K. J. (2016). Mechanisms of resistance to aminoglycoside antibiotics: overview and perspectives. *Med. Chem. Comm.* 7 11–27. 10.1039/C5MD00344J 26877861 PMC4752126

[B41] Georgopapadakou N. H. (1993). Penicillin-binding proteins and bacterial resistance to beta-lactams. *Antimicrob. Agents Chemother.* 37 2045–2053. 10.1128/AAC.37.10.2045 8257121 PMC192226

[B42] Girbau C. Guerra C. Martínez-Malaxetxebarria I. Alonso R. Fernández-Astorga A. (2015). Prevalence of ten putative virulence genes in the emerging foodborne pathogen *Arcobacter* isolated from food products. *Food Microbiol.* 52 146–149. 10.1016/j.fm.2015.07.015 26338128

[B43] Girbau C. Martinez-Malaxetxebarria I. Muruaga G. Carmona S. Alonso R. Fernandez-Astorga A. (2017). Study of biofilm formation ability of foodborne *Arcobacter butzleri* under different conditions. *J. Food. Prot.* 80 758–762. 10.4315/0362-028X.JFP-16-505 28358260

[B44] Gölz G. Alter T. Bereswill S. Heimesaat M. M. (2016). The immunopathogenic potential of *Arcobacter butzleri* - lessons from a meta-analysis of murine infection studies. *PLoS One* 11:e0159685. 10.1371/journal.pone.0159685 27438014 PMC4954699

[B45] Goris J. Konstantinidis K. T. Klappenbach J. A. Coenye T. Vandamme P. Tiedje J. M. (2007). DNA-DNA hybridization values and their relationship to whole-genome sequence similarities. *Int. J. Syst. Evol. Microbiol.* 57 81–91. 10.1099/ijs.0.64483-0 17220447

[B46] Groicher K. H. Firek B. A. Fujimoto D. F. Bayles K. W. (2000). The *Staphylococcus aureus* lrgAB operon modulates murein hydrolase activity and penicillin tolerance. *J. Bacteriol.* 182 1794–1801. 10.1128/JB.182.7.1794-1801.2000 10714982 PMC101860

[B47] Harms A. Fino C. Sørensen M. A. Semsey S. Gerdes K. (2017). Prophages and growth dynamics confound experimental results with antibiotic-tolerant persister cells. *mBio* 8:e01964-17. 10.1128/mBio.01964-17 29233898 PMC5727415

[B48] Hausdorf L. Neumann M. Bergmann I. Sobiella K. Mundt K. Fröhling A. (2013). Occurrence and genetic diversity of *Arcobacter* spp. in a spinach-processing plant and evaluation of two *Arcobacter*-specific quantitative PCR assay. *Syst. Appl. Microbiol.* 36 235–243. 10.1016/j.syapm.2013.02.003 23561260

[B49] Heimesaat M. M. Karadas G. Alutis M. Fischer A. Kühl A. A. Breithaupt A. (2015). Survey of small intestinal and systemic immune responses following murine *Arcobacter butzleri* infection. *Gut Pathog.* 7:28. 10.1186/s13099-015-0075-z 26483849 PMC4610047

[B50] ICMSF (2002). *Microorganisms in Foods. 7. Microbiological Testing in food safety management. International commission on Microbiological Specifications for Foods.* New York, NY: Kluwer Academic 10.1007/978-1-4615-0745-1

[B51] Jalava K. Rintala H. Ollgren J. Maunula L. Gomez-Alvarez V. Revez J. (2014). Novel microbiological and spatial statistical methods to improve strength of epidemiological evidence in a community-wide waterborne outbreak. *PLoS One* 9:e104713. 10.1371/journal.pone.0104713 25147923 PMC4141750

[B52] Javadi M. B. Katzenmeier G. (2016). The forgotten virulence factor: the ‘non-conventional’ hemolysin *tlya* and its role in *Helicobacter pylori* infection. *Curr. Microbiol.* 73 930–937. 10.1007/s00284-016-1141-6 27686341

[B53] Jiang Z. D. DuPont H. L. Brown E. L. Nandy R. K. Ramamurthy T. Sinha A. (2010). Microbial etiology of travelers’ diarrhea in Mexico, Guatemala, and India: importance of enterotoxigenic *Bacteroides fragilis* and *Arcobacter* species. *J. Clin. Microbiol.* 48 1417–1419. 10.1128/JCM.01709-09 20107088 PMC2849570

[B54] Juhas M. van der Meer J. R. Gaillard M. Harding R. M. Hood D. W. Crook D. W. (2009). Genomic islands: tools of bacterial horizontal gene transfer and evolution. *FEMS Microbiol. Rev.* 33 376–393. 10.1111/j.1574-6976.2008.00136.x 19178566 PMC2704930

[B55] Junecko J. M. Zielinska A. K. Mrak L. N. Ryan D. C. Graham J. W. Smeltzer M. S. (2012). Transcribing virulence in *Staphylococcus aureus*. *World J. Clin. Infect. Dis.* 2 63–76. 10.5495/wjcid.v2.i4.63

[B56] Kabeya H. Maruyama S. Morita Y. Ohsuga T. Ozawa S. Kobayashi Y. (2004). Prevalence of *Arcobacter* species in retail meats and antimicrobial susceptibility of the isolates in Japan. *Int. J. Food Microbiol.* 90 303–308. 10.1016/S0168-1605(03)00322-2 14751685

[B57] Kayman T. Abay S. Hizlisoy H. Atabay H. I. Diker K. S. Aydin F. (2012). Emerging pathogen *Arcobacter* spp. in acute gastroenteritis: molecular identification, antibiotic susceptibilities and genotyping of the isolated *Arcobacter*s. *J. Med. Microbiol.* 61 1439–1444. 10.1099/jmm.0.044594-0 22700547

[B58] Kim H. M. Hwang C. Y. Cho B. C. (2010). *Arcobacter marinus* sp. nov. *Int. J. Syst. Evol. Microbiol.* 60 531–536. 10.1099/ijs.0.007740-0 19654359

[B59] Kurincic M. Botteldoorn N. Herman L. Smole Mozina S. (2007). Mechanisms of erythromycin resistance of *Campylobacter* spp. isolated from food, animals and humans. *Int. J. Food Microbiol.* 120 186–189. 10.1016/j.ijfoodmicro.2007.03.012 17889390

[B60] Lau S. K. P. Woo P. C. Y. Teng J. Leung K. W. Yuen K. Y. (2002). Identification by 16S ribosomal RNA gene sequencing of *Arcobacter bacteraemia* in a patient with acute gangrenous appendicitis. *Mol. Pathol.* 55 182–185. 10.1136/mp.55.3.182 12032229 PMC1187171

[B61] Lee C. Agidi S. Marion J. W. Lee J. (2012). *Arcobacter* in Lake Erie beach waters: an emerging gastrointestinal pathogen linked with human-associated fecal contamination. *Appl. Environ. Microbiol.* 78 5511–5519. 10.1128/AEM.08009-11 22660704 PMC3406108

[B62] Lee M. Sousa M. C. (2014). Structural basis for substrate specificity in ArnB. A key enzyme in the polymyxin resistance pathway of Gram-negative bacteria. *Biochemistry.* 53 796–805. 10.1021/bi4015677 24460375 PMC3985747

[B63] Lee M. H. Choi C. (2013). Survival of *Arcobacter butzleri* in apple and pear purees. *J. Food Safety* 33 333–339. 10.1111/jfs.12057

[B64] Lehmann D. Alter T. Lehmann L. Uherkova S. Seidler T. Gölz G. (2015). Prevalence, virulence gene distribution and genetic diversity of *Arcobacter* in food samples in Germany. *Berl. Munch Tierarztl. Wochenschr.* 128 163–168. 25876277

[B65] Leoni F. Chierichetti S. Santarelli S. Talevi G. Masini L. Bartolini C. (2017). Occurrence of *Arcobacter* spp. and correlation with the bacterial indicator of faecal contamination *Escherichia coli* in bivalve molluscs from the Central Adriatic, Italy *Int. J. Food Microbiol.* 245 6–12. 10.1016/j.ijfoodmicro.2017.01.006 28113092

[B66] Levican A. Collado L. Figueras M. J. (2013). *Arcobacter cloacae* sp. nov. and *Arcobacter suis* sp. nov., two new species isolated from food and sewage. *Syst. Appl. Microbiol.* 36 22–27. 10.1016/j.syapm.2012.11.003 23265195

[B67] Levican A. Collado L. Yustes C. Aguilar C. Figueras M. J. (2014). Higher water temperature and incubation under aerobic and microaerobic conditions increase the recovery and diversity of *Arcobacter* spp. from shellfish. *Appl. Environ. Microbiol.* 80 385–391. 10.1128/AEM.03014-13 24185851 PMC3911005

[B68] Li L. G. Xia Y. Zhang T. (2017). Co-occurrence of antibiotic and metal resistance genes revealed in complete genome collection. *ISME J.* 11 651–662. 10.1038/ismej.2016.155 27959344 PMC5322307

[B69] Liu B. Pop M. (2009). ARDB-antibiotic resistance genes database. *Nucleic Acids Res.* 37 D443–D447. 10.1093/nar/gkn656 18832362 PMC2686595

[B70] Lupo A. Coyne S. Berendonk T. U. (2012). Origin and evolution of antibiotic resistance: the common mechanisms of emergence and spread in water bodies. *Front. Microbiol.* 3:18. 10.3389/fmicb.2012.00018 22303296 PMC3266646

[B71] Marchler-Bauer A. Bo Y. Han L. He J. Lanczycki C. J. Lu S. (2017). CDD/SPARCLE: functional classification of proteins via subfamily domain architectures. *Nucleic Acids Res.* 45 200–203. 10.1093/nar/gkw1129 27899674 PMC5210587

[B72] Matilla M. A. Krell T. (2018). The effect of bacterial chemotaxis on host infection and pathogenicity. *FEMS Microbiol. Rev.* 42:fux052. 10.1093/femsre/fux052 29069367

[B73] Matsumoto Y. Xu Q. Miyazaki S. Kaito C. Farr C. L. Axelrod H. L. (2010). Structure of a virulence regulatory factor CvfB reveals a novel winged helix RNA binding module. *Structure* 18 537–547. 10.1016/j.str.2010.02.007 20399190 PMC2858061

[B74] Meier-Kolthoff J. P. Auch A. F. Klenk H.-P. Göker M. (2013). Genome sequence-based species delimitation with confidence intervals and improved distance functions. *BMC Bioinformatics* 14:60. 10.1186/1471-2105-14-60 23432962 PMC3665452

[B75] Meier-Kolthoff J. P. Göker M. Klenk H.-P. (2014). Taxonomic use of DNA G+C content and DNA-DNA hybridization in the genomic age. *Int. J. Syst. Evol. Microbiol.* 64 352–356. 10.1099/ijs.0.056994-0 24505073

[B76] Merga J. Y. Royden A. Pandey A. K. Williams N. J. (2014). Arcobacter spp. isolated from untreated domestic effluent. *Lett. Appl. Microbiol.* 59 122–126. 10.1111/lam.12256 24666283

[B77] Miller W. G. Parker C. T. Rubenfield M. Mendz G. L. Wösten M. M. Ussery D. W. (2007). The complete genome sequence and analysis of the epsilonproteobacterium *Arcobacter butzleri*. *PLoS One* 2:e1358. 10.1371/journal.pone.0001358 18159241 PMC2147049

[B78] Minamino T. Macnab R. M. (2000). FliH, a soluble component of the type III flagellar export apparatus of *Salmonella*, forms a complex with FliI and inhibits its ATPase activity. *Mol. Microbiol.* 37 1494–1503. 10.1046/j.1365-2958.2000.02106.x 10998179

[B79] Mitchell A. L. Scheremetjew M. Denise H. Potter S. Tarkowska A. Qureshi M. (2017). EBI Metagenomics in 2017: enriching the analysis of microbial communities, from sequence reads to assemblies. *Nucleic Acids Res.* 46 D726–D735. 10.1093/nar/gkx967 29069476 PMC5753268

[B80] Mottola A. (2017). *Emerging Pathogen Arcobacter spp. in food: Occurrence and Genetic Diversity.* Ph.D. thesis, University of Bari “Aldo Moro”, Bari.

[B81] Mottola A. Bonerba E. Figueras M. J. Pérez-Cataluña A. Marchetti P. Serraino A. (2016). Occurrence of potentially pathogenic *Arcobacters* in shellfish. *Food Microbiol.* 57 23–27. 10.1016/j.fm.2015.12.010 27052698

[B82] Naas T. Oueslati S. Bonnin R. A. Dabos M. L. Zavala A. Dortet L. (2017). Beta-Lactamase DataBase (BLDB) – Structure and Function. *J. Enzyme Inhib. Med. Chem.* 32 917–919. 10.1080/14756366.2017.1344235 28719998 PMC6445328

[B83] Nicolosi D. Scalia M. Nicolosi V. M. Pignatello R. (2010). Encapsulation in fusogenic liposomes broadens the spectrum of action of vancomycin against Gram-negative bacteria. *Int. J. Antimicrob. Agent* 35 553–558. 10.1016/j.ijantimicag.2010.01.015 20219328

[B84] Nieva-Echevarria B. Martinez-Malaxetxebarria I. Girbau C. Alonso R. Fernández-Astorga A. (2013). Prevalence and genetic diversity of *Arcobacter* in food products in the north of Spain. *J. Food Prot.* 76 1447–1450. 10.4315/0362-028X.JFP-13-014 23905804

[B85] Nilsson I. Prathapam R. Grove K. Lapointe G. Six D. A. (2018). The sialic acid transporter NanT is necessary and sufficient for uptake of 3-deoxy-d-manno-oct-2-ulosonic acid (Kdo) and its azido analog in *Escherichia coli*. *Mol. Microbiol.* 110 204–218. 10.1111/mmi.14098 30076772

[B86] Oldfield N. J. Moran A. P. Millar L. A. Prendergast M. M. Ketley J. M. (2002). Characterization of the *Campylobacter jejuni* heptosyltransferase II gene, waaF, provides genetic evidence that extracellular polysaccharide is lipid A core independent. *J. Bacteriol.* 184 2100–2107. 10.1128/JB.184.8.2100-2107.2002 11914340 PMC134946

[B87] On S. L. W. Miller W. G. Houf K. Fox J. G. Vandamme P. (2017). Minimal standards for describing new species belonging to the families *Campylobacteraceae* and *Helicobacteraceae*: *Campylobacter*, *Arcobacter*, *Helicobacter* and *Wolinella* spp. *Int. J. Syst. Evol. Microbiol.* 67 5296–5311. 10.1099/ijsem.0.002255 29034857 PMC5845751

[B88] Otth L. Solís G. Wilson M. Fernández H. (2005). Susceptibility of *Arcobacter butzleri* to heavy metals. *Braz. J. Microbiol.* 36 286–288. 10.1590/S1517-83822005000300015

[B89] Otth L. Wilson M. Cancino R. Fernández H. (2004). In vitro susceptibility of *Arcobacter butzleri* to six antimicrobial drugs. *Arch. Med. Vet.* 36 207–210. 10.4067/S0301-732X2004000200012

[B90] Park S. Jung Y. T. Kim S. Yoon J. H. (2016). *Arcobacter acticola* sp. nov isolated from seawater on the East Sea in South Korea. *J. Microbiol.* 54 655–659. 10.1007/s12275-016-6268-4 27687227

[B91] Patyal A. Rathore R. S. Mohan H. V. Dhama K. Kumar A. (2011). Prevalence of *Arcobacter* spp. in humans, animals and foods of animal origin including sea food from India. *Transboundary Emerg. Dis.* 58 402–410. 10.1111/j.1865-1682.2011.01221.x 21477113

[B92] Pérez-Cataluña A. Salas-Massó N. Figueras M. J. (2018a). *Arcobacter canalis* sp. nov., isolated from a water canal contaminated with urban sewage. *Int. J. Syst. Evol. Microbiol.* 68 1258–1264. 10.1099/ijsem.0.002662 29488868

[B93] Pérez-Cataluña A. Salas-Massó N. Diéguez A. L. Balboa S. Lema A. Romalde J. L. (2018b). Corrigendum: Revisiting the taxonomy of the genus *Arcobacter*: getting order from the chaos. *Front. Microbiol.* 9:3123. 10.3389/fmicb.2018.03123 30622519 PMC6308300

[B94] Phillips C. A. (2001). *Arcobacters* as emerging human foodborne pathogens. *Food Control.* 12 1–6. 10.1016/S0956-7135(00)00024-4

[B95] Poole K. (2017). At the nexus of antibiotics and metals: the impact of Cu and Zn on antibiotic activity and resistance. *Trends Microbiol.* 25 820–832. 10.1016/j.tim.2017.04.010 28526548

[B96] Quintiliani R. Jr. Courvalin P. (1995). “Mechanisms of resistance to antimicrobial agents,” in *Manual of Clinical Microbiology*, 6th Edn, eds Murray P. R. Baron E. J. Pfaller M. A. Tenover F. C. Yolken R. H. (Washington, DC: ASM Press), 1319.

[B97] Rahimi E. (2014). Prevalence and antimicrobial resistance of *Arcobacter* species isolated from poultry meat in Iran. *Br. Poult. Sci.* 55 174–180. 10.1080/00071668.2013.878783 24404949

[B98] Ramees T. P. Dhama K. Karthik K. Rathore R. S. Kumar A. Saminathan M. (2017). *Arcobacter*: an emerging food-borne zoonotic pathogen, its public health concerns and advances in diagnosis and control – a comprehensive review. *Vet. Q.* 37 136–161. 10.1080/01652176.2017.1323355 28438095

[B99] Ramees T. P. Rathore R. S. Bagalkot P. S. Ravi Kumar G. V. P. P. S. Mohan H. V. Anoopraj R. (2014). Real- time PCR detection of *Arcobacter butzleri* and *Arcobacter cryaerophilus* in chicken meat samples. *J. Pure Appl. Microbiol.* 8 3165–3169. 10.1089/fpd.2009.0368 19899959

[B100] Rathlavath S. Kohli V. Singh A. S. Lekshmi M. Tripathi G. Kumar S. (2017). Virulence genotypes and antimicrobial susceptibility patterns of *Arcobacter butzleri* isolated from seafood and its environment. *Int. J. Food Microbiol.* 263 32–37. 10.1016/j.ijfoodmicro.2017.10.005 29028568

[B101] Richards V. P. Lefébure T. Pavinski Bitar P. D. Stanhope M. J. (2013). Comparative characterization of the virulence gene clusters (lipooligosaccharide [LOS] and capsular polysaccharide [CPS]) for *Campylobacter coli*, *Campylobacter jejuni* subsp. *jejuni* and related *Campylobacter* species. *Infect. Genet. Evol.* 14 200–213. 10.1016/j.meegid.2012.12.010 23279811 PMC3622452

[B102] Riesenberg A. Frömke C. Stingl K. Feßler A. T. Gölz G. Glocker E. O. (2017). Antimicrobial susceptibility testing of *Arcobacter butzleri*: development and application of a new protocol for broth microdilution. *J. Antimicrob. Chemother.* 72 2769–2774. 10.1093/jac/dkx211 29091194

[B103] Rodriguez-R L. M. Konstantinidis K. T. (2016). The enveomics collection: a toolbox for specialized analyses of microbial genomes and metagenomes. *PeerJ. Preprints* 4:e1900v1.

[B104] Rossmann F. M. Beeby M. (2018). Insights into the evolution of bacterial flagellar motors from high-throughput in situ electron cryotomography and subtomogram averaging. *Acta Crystallogr. D. Struct. Biol.* 74 585–594. 10.1107/S2059798318007945 29872008 PMC6096493

[B105] Rovetto F. Carlier A. Van den Abeele A. M. Illeghems K. Van Nieuwerburgh F. Cocolin L. (2017). Characterization of the emerging zoonotic pathogen *Arcobacter thereius* by whole genome sequencing and comparative genomics. *PLoS One* 12:e0180493. 10.1371/journal.pone.0180493 28671965 PMC5495459

[B106] Shah A. H. Saleha A. A Murugaiyah M. Zunita Z. Memon A. A. (2012). Prevalence and distribution of *Arcobacter* spp. in raw milk and retail raw beef. *J. Food Protect.* 75 1474–1478. 10.4315/0362-028X.JFP-11-487 22856572

[B107] Scanlon K. A. Cagney C. Walsh D. McNulty D. Carroll A. McNamara E. B. (2013). Occurrence and characteristics of fastidious *Campylobacteraceae* species in porcine samples. *Int. J. Food Microbiol.* 163 6–13. 10.1016/j.ijfoodmicro.2013.02.004 23474652

[B108] Schauer Weissfeld A. (2014). Infections from eating raw or undercooked seafood. *Clin. Microbiol. Newsl.* 36 17–21. 10.1016/j.clinmicnews.2014.01.004

[B109] Seiler C. Berendonk T. U. (2012). Heavy metal driven co-selection of antibiotic resistance in soil and water bodies impacted by agriculture and aquaculture. *Front. Microbiol.* 3:399. 10.3389/fmicb.2012.00399 23248620 PMC3522115

[B110] Šilha D. Pejchalová M. Šilhová L. (2017). Susceptibility to 18 drugs and multidrug resistance of *Arcobacter* isolates from different sources within the Czech Republic. *J. Glob. Antimicrob. Resist.* 9:74e77. 10.1016/j.jgar.2017.01.006 28400212

[B111] Soma S. M. Srinivasa R. T. Bindu K. C. H. Subramanyam K. V. Mohammad S. N. (2017). Antibiogram of *Arcobacter* species isolated from animals, foods of animal origin and humans in Andhra Pradesh, India. *Int. J. Sci. Environ. Tech.* 6 1260–1269. 10.14202/vetworld.2017.716-720

[B112] Talay F. Molva C. Atabay H. I. (2016). Isolation and identification of *Arcobacter* species from environmental and drinking water samples. *Folia Microbiol.* 61 479–484. 10.1007/s12223-016-0460-0 27106697

[B113] Tatusova T. DiCuccio M. Badretdin A. Chetvernin V. Nawrocki E. P. Zaslavsky L. (2016). NCBI prokaryotic genome annotation pipeline. *Nucleic Acids Res.* 44 6614–6624. 10.1093/nar/gkw569 27342282 PMC5001611

[B114] The UniProt Consortium (2017). UniProt: the universal protein knowledgebase. *Nucleic Acids Res.* 45 D158–D169. 10.1093/nar/gkw1099 27899622 PMC5210571

[B115] Tiwari S. Jamal S. B. Hassan S. S. Carvalho P. V. S. D. Almeida S. Barh D. (2017). Two-component signal transduction systems of pathogenic bacteria as targets for antimicrobial therapy: an overview. *Front. Microbiol.* 8:1878. 10.3389/fmicb.2017.01878 29067003 PMC5641358

[B116] Van den Abeele A. M. Vogelaers D. Van Hende J. Houf K. (2014). Prevalence of *Arcobacter* species among humans, Belgium, 2008–2013. *Emerg. Infect. Dis.* 20 1731–1734. 10.3201/eid2010.140433 25271569 PMC4193277

[B117] Van den Abeele A. M. Vogelaers D. Vanleare E. Houf K. (2016). Antimicrobial susceptibility testing of *Arcobacter butzleri* and *Arcobacter cryaerophilus* strains isolated from Belgian patients. *J. Antimicrob. Chemother.* 71 1241–1244. 10.1093/jac/dkv483 26851610

[B118] Vandamme P. Vancanneyt M. Pot B. Mels L. Hoste B. Dewettinck D. (1992). Polyphasic taxonomic study of the emended genus *Arcobacter* with *Arcobacter butzleri* comb. nov. and *Arcobacter skirrowii* sp. nov., an *Aerotolerant bacterium* isolated from veterinary specimens. *Int. J. Syst. Bacteriol.* 42 344–345. 10.1099/00207713-42-3-344 1503968

[B119] Vandenberg O. Houf K. Douat N. Vlaes L. Retore P. Butzler J. P. (2006). Antimicrobial susceptibility of clinical isolates of non-jejuni/coli *Campylobacters* and *Arcobacters* from Belgium. *J. Antimicrob. Chemot.* 57 908–913. 10.1093/jac/dkl080 16533825

[B120] Vicente-Martins S. Oleastro M. Domingues F. C. Ferreira S. (2018). Arcobacter spp. at retail food from Portugal: prevalence, genotyping and antibiotics resistance. *Food Control.* 85 107–112. 10.1016/j.foodcont.2017.09.024

[B121] Waite D. W. Vanwonterghem I. Rinke C. Parks D. H. Zhang Y. Takai K. (2017). Comparative genomic analysis of the class *Epsilonproteobacteria* and proposed reclassification to *Epsilonbacteraeota* (phyl. nov.). *Front. Microbiol.* 8:682 10.3389/fmicb.2017.00682 PMC5401914 28484436

[B122] Waite D. W. Vanwonterghem I. Rinke C. Parks D. H. Zhang Y. Takai K. (2018). Erratum: Addendum: comparative genomic analysis of the Class *Epsilonproteobacteria* and proposed reclassification to *Epsilonbacteraeota* (phyl. nov.). *Front. Microbiol.* 18:772. 10.3389/fmicb.2018.00772 29720974 PMC5915535

[B123] Webb A. L. Boras V. F. Kruczkiewicz P. Selinger L. B. Taboada E. N. Inglis G. D. (2016). Comparative detection and quantification of *Arcobacter butzleri* in stools from diarrheic and non-diarrheic human beings in southwestern Alberta, Canada. *J. Clinic. Microbiol.* 54 1082–1088. 10.1128/JCM.03202-15 26865686 PMC4809925

[B124] Wilson D. N. (2014). Ribosome-targeting antibiotics and mechanisms of bacterial resistance. *Nat. Rev. Microbiol.* 12 35–48. 10.1038/nrmicro3155 24336183

[B125] Wu D. Dolfing J. Xie B. (2018). Bacterial perspectives on the dissemination of antibiotic resistance genes in domestic wastewater bio-treatment systems: beneficiary to victim. *Appl. Microbiol. Biotechnol.* 102 597–604. 10.1007/s00253-017-8665-y 29198067

[B126] Yesilmen S. Vural A. Erkan M. E. Yildirim I. H. (2014). Prevalence and antimicrobial susceptibility of *Arcobacter* species in cow milk, water buffalo milk and fresh village cheese. *Int. J. Food Microbiol.* 188 11–14. 10.1016/j.ijfoodmicro.2014.07.006 25064812

[B127] Yu Z. Gunn L. Wall P. Fanning S. (2017). Antimicrobial resistance and its association with tolerance to heavy metals in agriculture production. *Food Microbiol.* 64 23–32. 10.1016/j.fm.2016.12.009 28213031

[B128] Zacharow I. Bystroń J. Wałecka-Zacharska E. Podkowik M. Bania J. (2015a). Genetic diversity and incidence of virulence-associated genes of *Arcobacter* *butzleri* and *Arcobacter* *cryaerophilus* isolates from pork, beef, and chicken meat in Poland. *Biomed. Res. Int.* 2015:ID956507. 10.1155/2015/956507 26539546 PMC4619883

[B129] Zacharow I. Bystroń J. Wałecka-Zacharska E. Podkowik M. Bania J. (2015b). Prevalence and antimicrobial resistance of *Arcobacter butzleri* and *Arcobacter cryaerophilus* isolates from retail meat in lower Silesia region, Poland. *Pol. J. Vet. Sci.* 18 63–69. 25928911 10.1515/pjvs-2015-0008

[B130] Zhu S. Nishikino T. Hu B. Kojima S. Homma M. Liu J. (2017). Molecular architecture of the sheathed polar flagellum in *Vibrio alginolyticus*. *Proc. Natl. Acad. Sci. U.S.A.* 114 10966–10971. 10.1073/pnas.1712489114 28973904 PMC5642721

