# Supplementary material for: Genomic Characterization of Arcobacter butzleri Isolated From Shellfish: Novel Insight Into Antibiotic Resistance and Virulence Determinants
Source: Front Microbiol. 2019 Apr 16;10:670. doi: 10.3389/fmicb.2019.00670 (PMC6477937; doi:10.3389/fmicb.2019.00670)
Supplement: Supplementary file 1 [file Table_1.DOCX]

| **Table S1**. Strain used in this study, source of isolation and accession numbers of available genomes or reference Sequences | | | |
| --- | --- | --- | --- |
| **Species** | **Strain** | **Source** | **Accession n. /reference sequence** |
| *A. anaerophilus* | IR-1 | Utsira aquifer | NZ_JXXG00 |
| *A. butzleri* | 55 | mussels | QXMK00000000 |
| *A. butzleri* | 6V | clams | QXNB0000000 |
| *A. butzleri* | RM4018 | human patient with gastroenteritis | NC_009850 |
| *A. butzleri* | NTCT 12481 | faeces / diarrhoea | NZ_LT906455.1 |
| *A. butzleri* | 7h1h | faeces of a healthy dairy cow | NC_021878 |
| *A. butzleri* | ED-1 | microbial fuel cell | NC_017187 |
| *A. butzleri* | JV22 | human gastrointestinal tract | AEPT00000000 |
| *A. canalis* | F138-33 | Oyster PNC | NZ_NWVW01 |
| *A. cibarius* | LMG 21996 | broiler, skin | NZ_JABW00000000.1 |
| *A. cryaerophilus* | L406 | water | NZ_LRUV00000000.1 |
| *A. defluvii* | L | microbial fuel cell | NC_017192 |
| *A. faecis* | AF1078 | human sewage tank | NZ_JARS00000000 |
| *A. halophilus* | DSM 18005 | hypersaline lagoon | NZ_NXIF00 |
| *A. lanthieri* | AF1440 | pig faecal storage tank | JARU00000000 |
| *A. lekithochrous* | LFT 1.7 | scallop larvae | NZ_MKCO00 |
| *A. marinus* | SH-4D_Col1 | unknown | NZ_FUYO00 |
| *A. molluscorum* | CECT 7696 | mussels | NZ_NXFY00000000 |
| *A. nitrofigilis* | DSM 7299 | marshland plant | NC_014166 |
| *A. porcinus* | 213 | slaughter house | LCSK01000001.1 |
| *A. skirrowii* | L403 | water | NZ_LRUX00000000.1 |
| *A. thereius* | 24486 | aborted pig foetus | NZ_LLKQ00000000.1 |
